# Absolute Aerobic Capacity Predominates over Body Composition and Regional Anthropometry in Explaining Maximal Fat Oxidation in National-Team Collegiate Athletes: A Cross-Sectional Study

**DOI:** 10.3390/jfmk11030349

**Published:** 2026-09-03

**Authors:** Carlos Abraham Herrera-Amante, Brenda Cecilia Jaime-González, Diego A. Bonilla, Rodrigo Yáñez-Sepúlveda, José Raúl Hoyos-Flores, César Octavio Ramos-García, José Francisco López-Gil

**Affiliations:** 1 Nutritional Assessment and Nutritional Care Laboratory, Division of Health Sciences, Tonala University Center, University of Guadalajara, Tonala 45425, Mexico; carlos.amante@academicos.udg.mx (C.A.H.-A.); jaimebrendac@gmail.com (B.C.J.-G.); octavio.ramos@academicos.udg.mx (C.O.R.-G.); 2 Ibero-American Network of Researchers in Applied Anthropometry, 04120 Almeria, Spain; dabonilla@dbss.pro (D.A.B.); jose.hoyosfl@uanl.edu.mx (J.R.H.-F.); 3 Research Group in Sports Nutrition (DBSS-Nut), Dynamical Business & Science Society—DBSS International SAS, Bogotá 110311, Colombia; 4 Hologenomiks Research Group, Department of Genetics, Physical Anthropology and Animal Physiology, University of the Basque Country (UPV/EHU), 48940 Leioa, Spain; 5 Faculty of Education and Humanities, School of Sport Sciences, Universidad Andres Bello, Viña del Mar 2520000, Chile; rodrigo.yanez.s@unab.cl; 6 Department of Health Research, Icen Cognis, 38370 La Matanza de Acentejo, Spain; 7 Faculty of Sports Organization, Autonomous University of Nuevo Leon, San Nicolas de los Garza 66455, Mexico; 8 School of Medicine, Universidad Espiritu Santo, Samborondon 092301, Ecuador; 9 Faculty of Health Sciences, Universidad Tecnológica Atlántico Mediterráneo—UTAMED, 29590 Málaga, Spain

**Keywords:** kinanthropometry, corrected girths, body composition, FATmax, maximal fat oxidation, indirect calorimetry

## Abstract

**Background:** Maximal fat oxidation (MFO) is the highest rate of fat oxidation attained during incremental exercise, with cardiorespiratory fitness being its primary correlate. How body composition and regional anthropometry explain MFO variability remains poorly understood. While fat-free mass is a known correlate, regional morphology, such as skinfold-corrected girth, has been proposed to capture local muscle mass contributions. However, MFO is an absolute rate (g·min^−1^) whereas peak oxygen uptake (VO_2_peak, typically mL·kg^−1^·min^−1^) is conventionally reported relative to body mass. The statistical consequences of this dimensional mismatch, and the question of whether regional girths independently account for MFO variance or merely reflect body size artefacts, remain unexamined. **Methods:** Forty-three national-team collegiate athletes (21 men, 22 women) underwent a maximal incremental treadmill test with breath-by-breath indirect calorimetry, dual-energy X-ray absorptiometry (DXA), bioelectrical impedance analysis and a full International Society for the Advancement of Kinanthropometry (ISAK) anthropometric profile including six skinfold-corrected girths. The primary model regressed absolute MFO on absolute peak oxygen uptake (VO_2_peak, L·min^−1^). Sensitivity models added corrected girths, fat-free mass, sex, stature and body mass. Internal validity was assessed by leave-one-out cross-validation (*Q*^2^) and 5000-sample bootstrapping, with Benjamini–Hochberg control of the false discovery rate. **Results:** Absolute VO_2_peak explained 60.4% of the variance in MFO (*Q*^2^ = 0.563; *β* = 0.191 g·min^−1^ per L·min^−1^, 95% CI 0.142 to 0.240; bootstrap CI 0.133 to 0.227). Adding corrected calf girth changed the explained variance by 0.006 (*p* = 0.424) and reduced *Q*^2^ to 0.541. No corrected girth survived the false-discovery-rate control, and fat-free mass added nothing (*p* = 0.268). When aerobic capacity was instead expressed per kilogram of body mass, model fit was substantially worse (*R*^2^ = 0.415) and corrected calf girth appeared to be strongly and independently associated with MFO (*p* < 0.001). **Conclusions:** In trained athletes, absolute aerobic capacity explains MFO variance, whereas regional anthropometry and body composition provide no incremental information. Normalising predictor variables by body mass creates statistical artefacts that falsely attribute explanatory power to structural girths. Future studies with larger sample sizes and diverse athletic populations are needed to confirm these findings across different training statuses and metabolic profiles.

## 1. Introduction

Maximal fat oxidation (MFO) is the highest rate of fat oxidation attained during a graded exercise test, and the intensity at which it occurs is termed FATmax [1,2]. In athletic populations, MFO has attracted interest because a greater capacity to oxidise lipids at submaximal intensities may spare endogenous carbohydrate stores during prolonged exercise [3,4]. In clinical and public-health contexts, MFO has been interpreted as one accessible expression of the capacity to switch between substrates, although it captures only the exercise-related component of that construct and not the broader phenomenon [5,6], and lower values have been related to a less favourable cardiometabolic profile in young adults [7].

The determinants of between-individual variability in MFO are incompletely understood. Cardiorespiratory fitness is the most consistently reported correlate [8,9,10], but sex [8,11], adiposity [12,13], habitual dietary intake [14], training status [15] and genetic variation [16,17,18] have all been implicated, and effect estimates differ considerably across studies [19]. Methodological heterogeneity in how MFO is elicited and computed contributes substantially to this dispersion [1,20].

Understanding the extent to which body composition and regional anthropometry explain individual variations in MFO remains a key question in sports science [21]. While whole-body mass and fat-free mass are known correlates, regional morphology, such as skinfold-corrected girth [22,23], may provide deeper insights into the contribution of local muscle mass to lipid oxidation capacity [23]. Determining whether these structural variables independently account for MFO variance, or merely reflect body size artefacts, is essential for evaluating their practical utility in athletic profiling.

However, an unresolved specification issue complicates the interpretation of such associations. MFO is an absolute rate expressed in grams per minute, whereas peak oxygen uptake (VO_2_peak) is conventionally reported per kilogram of body mass. Regressing an absolute outcome on a size-normalised predictor leaves a component of body size unmodelled, and any additional variable that carries body-size information, such as a limb girth, can then appear to contribute independently. Tanner described this class of artefact in relation to per-weight and per-surface-area standards more than seven decades ago [24], yet it has rarely been examined explicitly in the MFO literature.

The present study therefore had two aims. The primary aim was to quantify the association between aerobic capacity and MFO using a dimensionally consistent specification, in which an absolute outcome is modelled on an absolute predictor. The secondary aim was to test whether skinfold-corrected girths, fat-free mass (FFM) or other body-size descriptors contribute information about MFO beyond that specification, and to determine whether the choice between an absolute and a size-normalised expression of aerobic capacity materially changes the conclusions drawn.

## 2. Materials and Methods

### 2.1. Study Design and Analytical Strategy

**Figure 1:**
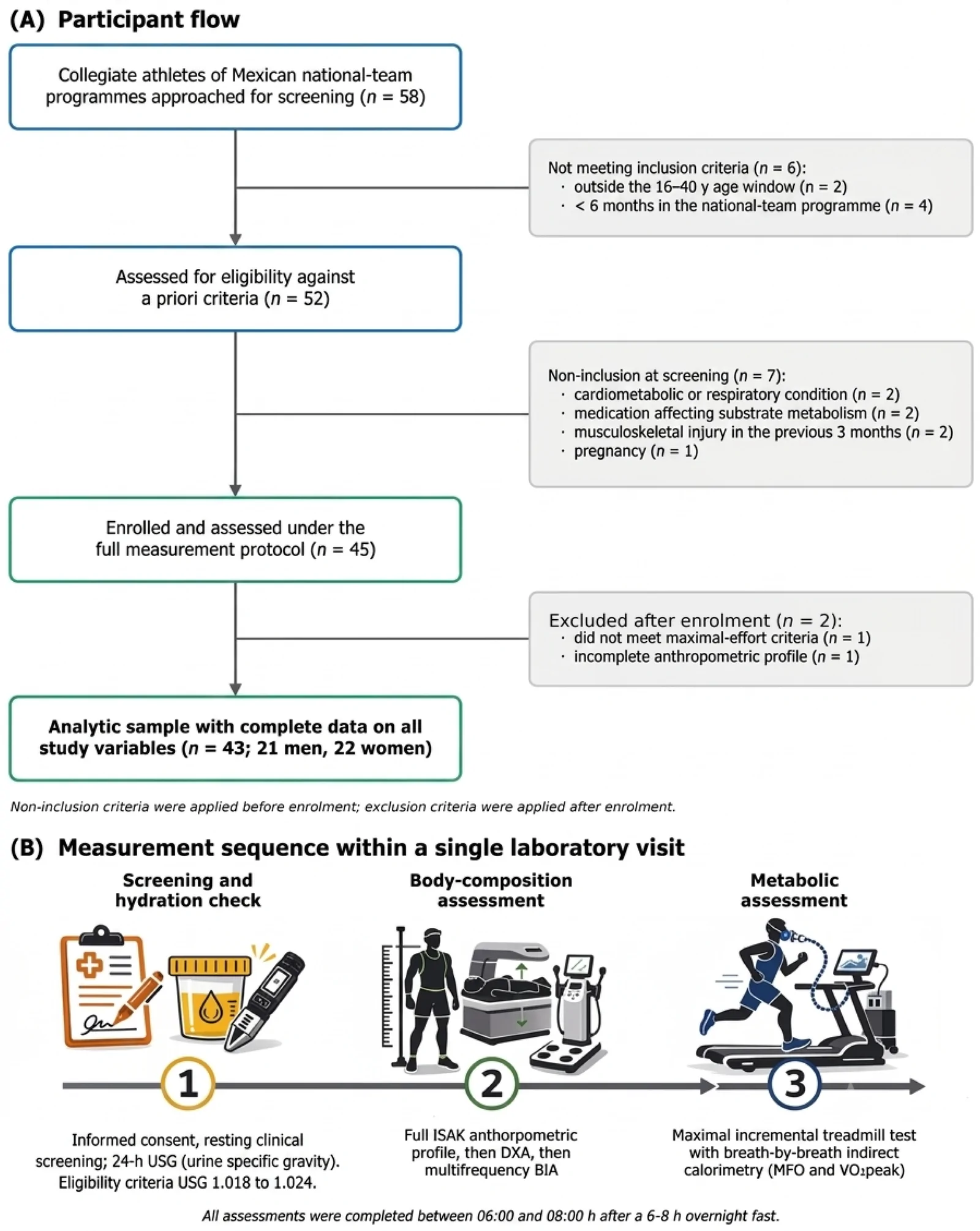
Study design, participant flow and measurement sequence. (**A**) Flow of collegiate athletes from Mexican national-team programmes through screening, enrolment and analysis; non-inclusion criteria were applied before enrolment and exclusion criteria after enrolment. (**B**) The three sequential phases of the protocol, all completed within a single laboratory visit between 06:00 and 08:00 h after a 6–8 h overnight fast: (1) informed consent, resting clinical screening and verification of hydration status by urine specific gravity (24 h USG; eligibility window 1.018–1.024, a value outside the window on the scheduled day led to rescheduling rather than to permanent exclusion, see Section 2.3); (2) body-composition assessment by a full ISAK anthropometric profile, dual-energy X-ray absorptiometry (DXA) and multifrequency bioelectrical impedance analysis (BIA); and (3) determination of maximal fat oxidation (MFO) and peak oxygen uptake (VO_2_peak) during a maximal incremental treadmill test with breath-by-breath indirect calorimetry. ISAK, International Society for the Advancement of Kinanthropometry.

This was a cross-sectional, observational study reported in accordance with the Strengthening Reporting of Observational Studies in Epidemiology (STROBE) statement [25,26]. A single analytical hierarchy was defined before the analyses reported here were performed. One primary model was specified on physiological and dimensional grounds: absolute MFO (g·min^−1^) regressed on absolute VO_2_peak (L·min^−1^). Fat oxidation during exercise is supported by whole-body oxidative metabolism, so the quantity of oxygen consumed per unit time is the theoretically appropriate scale on which to model a mass of substrate oxidised per unit time. All further models were designated in advance as sensitivity or exploratory analyses, and no automated variable-selection procedure was used at any stage, in line with current recommendations [27]. The participant flow and the measurement sequence are summarised in Figure 1.

### 2.2. Setting and Ethics

Assessments were conducted between 1 June and 30 August 2025 at the Nutritional Status Assessment and Care Laboratory (LECEN) of the Tonala University Center, University of Guadalajara, Tonala, Mexico, under controlled conditions (20–25 °C; 50–60% relative humidity). The study was conducted in accordance with the Declaration of Helsinki [28] and the Regulations of the General Health Law on Health Research of Mexico [29], and was approved by the Research Ethics Committee of the University of Guadalajara (CEI-02-2024-07, approved 11 July 2024). Informed consent was obtained in accordance with Articles 20–22 of those Regulations.

### 2.3. Participants and Eligibility

Forty-three collegiate athletes who were active members of Mexican national teams and had represented Mexico in international competition were recruited by institutional invitation (21 men, 22 women). All corresponded to Tier 4 (“Elite/International Level”) of the participant classification framework of McKay et al. [30]. To contextualise the heterogeneity of the sample, sports were grouped descriptively according to the predominant energy system as oxidative (*n* = 14; triathlon, sailing, 5000 m running, mountain biking), glycolytic (*n* = 18; 400–800 m track events, 400 m hurdles, Brazilian jiu-jitsu, wushu, taekwondo, karate), phosphagen (*n* = 4; weightlifting, 100–200 m sprint events) and intermittent (*n* = 7; soccer, volleyball, flag football, basketball). All evaluations were performed during the general preparation phase, outside competitive and transition periods.

Inclusion criteria were: (i) active membership of a Mexican national team for at least six months, with international competitive experience; (ii) age between 16 and 40 years; (iii) apparently healthy status; (iv) a 24 h urine specific gravity (USG) within the pre-defined euhydration window of 1.018–1.024 on the day of testing; and (v) written informed consent, together with written parental or guardian consent and participant assent for those under 18 years of age.

Non-inclusion criteria, that is, conditions assessed at screening and applied before enrolment, were: (i) comorbidities capable of influencing the study variables (e.g., hypertension, hyperthyroidism or cardiovascular disease); (ii) use of medications or stimulants (e.g., caffeine or diuretics) within 48 h before testing; (iii) pregnancy; and (iv) musculoskeletal injury within the previous three months. Exclusion criteria, that is, conditions applied after enrolment and leading to withdrawal of an already-enrolled participant, were the occurrence of injury or illness before or during the study period, failure to satisfy the maximal-effort criteria during the incremental test, and an incomplete anthropometric profile. The two categories are kept distinct because they carry different implications for selection: non-inclusion criteria are ascertained before enrolment and therefore define the source population, whereas exclusion criteria are applied after enrolment and may introduce attrition. No participant was eliminated because of injury or illness; two enrolled participants were eliminated, one who did not satisfy the maximal-effort criteria and one with an incomplete anthropometric profile (Figure 1A).

Participant age ranged from 16 to 39 years (median 21 years; inclusion criteria permitted up to 40 years). Participation was strictly voluntary and unrelated to team selection, competition eligibility, or academic evaluation. No incentives were offered, and participants could withdraw at any time. All individual-level results were delivered exclusively to the athletes, leaving any further sharing to their discretion. For adult participants, coaches were absent during consent and testing, and received no data from the research team. For minor participants, written informed consent was obtained from a parent or legal guardian alongside written assent from the athlete; to safeguard the minors, parents or legal guardians were required to be present during testing, and coaches were permitted to attend only upon request by the athlete or their legal guardian.

Before the maximal test, all participants completed a health-history questionnaire and a resting clinical screening (resting blood pressure, resting heart rate and auscultation) performed by the laboratory’s medical staff, and were cleared for maximal exertion in the absence of absolute contraindications to cardiopulmonary exercise testing [31]. Pregnancy was screened by direct interview; no biochemical test was performed. All maximal tests were supervised by at least two trained staff members, with continuous heart-rate and symptom monitoring, an emergency protocol, an automated external defibrillator and a first-aid kit in the testing room, and a defined referral pathway to the university health service. No adverse events occurred.

Hydration status was verified on arrival (06:00–08:00 h), before any other measurement, from a single mid-stream urine sample analysed in duplicate with a calibrated digital refractometer (ATAGO^®^ PAL-10S; ATAGO CO., Ltd., Minato, Tokyo, Japan). The window of 1.018–1.024 was taken from reference values for euhydrated, physically active adults [32] and has been applied in previous work from this laboratory [33]; its lower bound excludes an excessively dilute first-void sample and its upper bound excludes hypohydration. Because euhydration was an inclusion requirement rather than a post-enrolment criterion, participants whose 24 h USG fell outside the window were rescheduled and reassessed after standardised rehydration instruction rather than excluded. This occurred in two participants, both of whom were subsequently tested within the window. There were no missing values for any variable used in these analyses, so the denominator was *N* = 43 throughout. The complete participation flow, from the athletes approached for screening to the analytic sample, is reported in Figure 1A and was compiled from the recruitment and screening records held by the laboratory.

### 2.4. Anthropometry

Participants attended wearing tight-fitting clothing and without metallic objects. A complete anthropometric profile was constructed following the protocol of the International Society for the Advancement of Kinanthropometry (ISAK) [21], with the addition of the submandibular skinfold as described previously [22]. Body mass was recorded with a digital scale accurate to 100 g (Seca^®^ 874, Hamburg, Germany) and stretch stature with a portable stadiometer accurate to 1 mm (Seca^®^ 213). Skinfolds were measured with a Slim-Guide^®^ skinfold calliper (Creative Health Products, Plymouth, MI, USA), previously verified with a laboratory load cell to confirm a closing pressure between 9.2 g·mm^−2^ (20 mm opening) and 10.1 g·mm^−2^ (40 mm opening). Girths were measured with a non-extensible metallic tape 0.7 mm thick, and lengths and breadths with professional anthropometric equipment accurate to 1 mm (SmartMet Kinanthropometric Assessment^®^, Jalisco, Mexico).

All measurements in all participants were obtained by a single ISAK Level 2 certified anthropometrist; inter-observer error therefore does not apply to this dataset. Every site was measured in duplicate, with a third measurement and retention of the median when duplicates differed by more than 5% for skinfolds or 1% for girths, lengths and breadths. The intra-observer technical error of measurement, computed for this operator during the study period, was <3.2% for skinfolds and <0.86% for all other measurements.

Corrected girths were calculated by subtracting the corresponding skinfold thickness (converted to centimetres) multiplied by π from the respective girth [22]: submandibular skinfold for neck, triceps for arm, subscapular for chest, supraspinale for waist, anterior thigh for thigh and medial calf for calf. Corrected girths are indirect indicators of the limb tissue remaining once the estimated subcutaneous adipose layer has been removed, and they do not measure muscle mass, muscle cross-sectional area, muscle quality, fibre-type composition or oxidative capacity. They are treated as anthropometric surrogates throughout.

### 2.5. Body Composition

Body composition was assessed by dual-energy X-ray absorptiometry (DXA) with a Lunar iDXA system (General Electric^®^, Madison, WI, USA), with daily calibration according to the manufacturer’s recommendations [34] and standardised acquisition and interpretation procedures [35,36,37]. Scans were acquired and analysed with the GE enCORE platform version 18 in standard scan mode, with participants supine, centred, palms flat and feet secured with the manufacturer’s strap. Region-of-interest cut lines were visually inspected and manually adjusted following the anatomical criteria of the 2023 Adult Official Positions of the International Society for Clinical Densitometry [35]. Positioning, acquisition and analysis were performed by the same trained technician throughout. A pilot repositioning test–retest assessment in 30 individuals, performed by the same operator on the same unit with the same protocol, yielded coefficients of variation of 0.73% for total fat mass and 0.86% for body fat percentage, consistent with published precision for this system [38].

As a complementary method, multifrequency bioelectrical impedance analysis was performed with an InBody 720 device (InBody Co^®^, Cerritos, CA, USA), which shows good agreement with DXA [39]; data were exported with LookinBody version 3.0 and FFM was derived as body mass minus fat mass. Standardisation comprised a 6–8 h overnight fast, bladder voiding immediately before measurement, abstention from exercise for 12 h and from strenuous exercise for 24 h, abstention from alcohol and caffeine, and 5 min of quiet standing before the scan. DXA was designated the criterion method; where DXA- and BIA-derived estimates disagreed, no averaging or substitution was performed, both sets of variables were reported separately, and only DXA-derived variables entered the regression models. This is a reporting convention, not a validation claim: agreement between methods was not formally quantified in this dataset.

### 2.6. Maximal Fat Oxidation, VO_2_peak and Resting Heart Rate

Ergospirometry was performed with a breath-by-breath indirect calorimetry system (PNOĒ^®^, ENDO Medical, Palo Alto, CA, USA; serial number PNOĒ 2116-393). Participants completed a familiarisation session beforehand [31]. Hardware accuracy was verified before the study period with an automated pulmonary simulator across tidal volumes of 1.5–4.0 L and breathing frequencies of 27–35 breaths·min^−1^. Pre-test calibration was verified daily following the manufacturer’s automated protocol (ambient-air gas-sensor calibration at 20.93% O_2_ and 0.03% CO_2_, and flow/volume calibration with a 3 L syringe). The device does not use local or user-selectable software versions; data were exported in standard breath-by-breath format, with the moving-average filter as the sole user-defined processing parameter. A 3-breath moving-average filter was applied directly within the online platform (PNOĒ Analytics) to eliminate transient artefacts. Data were then time-averaged, and the mean values of the final 60 s of each 3 min stage were used to compute substrate oxidation rates, consistent with the steady-state assumption required for indirect calorimetry [40,41]. No additional manual breath exclusion was performed.

Participants arrived after a 6–8 h overnight fast, having abstained from exercise for at least 12 h and from alcohol and stimulant-containing products for at least 48 h. The postabsorptive state is reached approximately 4–6 h after the last meal, once the acute postprandial suppression of lipolysis has dissipated [40,41], and published MFO protocols show substantial heterogeneity in fasting duration, including windows of 4–8 h [1]. Extending the fast to 10–12 h before an early-morning maximal treadmill test in this population was judged likely to compromise hepatic glycogen availability, effort tolerance and adherence; the consequences of this choice are addressed in the limitations. All assessments were performed between 06:00 and 08:00 h to limit circadian influences. Total pre-test dietary intake was not standardised or quantified, and habitual training load in the preceding days was not recorded prospectively.

Female athletes were assessed during the early follicular phase (self-reported days 1–5 of menstruation) to limit sex-hormone-related variation in substrate metabolism [42]; no participant was using hormonal contraception. Cycle phase was confirmed by self-report only, without hormonal assay.

The incremental treadmill test (Horizon Fitness^®^ T202-06; Johnson Health Tech, Ho Chi Minh City, Vietnam) used the speed-and-stage structure described by Pugh [43]. That source is a study of oxygen intake in track and treadmill running and is used here only as the origin of the incremental structure; it is not, and is not presented as, a validated MFO determination protocol. Stage duration and the computation of oxidation rates followed current methodological recommendations for MFO assessment [1,20]. The test began at 6 km·h^−1^ with increments of 2 km·h^−1^ every 3 min until volitional exhaustion. MFO (g·min^−1^) was estimated with the stoichiometric equations of Péronnet and Massicotte [44]. Although the test continued to exhaustion in order to determine VO_2_peak, MFO was identified exclusively from the submaximal stages, before the shift towards predominant carbohydrate oxidation. MFO was defined as the highest stage-mean fat oxidation rate across submaximal stages; when two adjacent stages yielded identical or minimally different rates, the stage of lower intensity was retained, a rule defined before analysis and applied uniformly. The coarse resolution of this stage structure, and its consequences for the estimated MFO value, are addressed in the limitations.

VO_2_peak was accepted after volitional exhaustion when at least two secondary criteria were satisfied: respiratory exchange ratio ≥ 1.10; attainment of ≥95% of age-predicted maximal heart rate; or a rating of perceived exertion ≥ 9 on the Borg 1–10 scale. A plateau in oxygen uptake was not used as a criterion, so the value reported is a peak rather than a verified maximum and is labelled VO_2_peak throughout. Absolute VO_2_peak (L·min^−1^) is the primary predictor in all models; the body-mass-normalised value (mL·kg^−1^·min^−1^) is reported for descriptive comparability and is examined explicitly as an alternative specification. Heart rate was monitored continuously with a Polar H10 monitor (Polar Electro^®^ Oy, Kempele, Finland). Resting heart rate was recorded on arrival, before any other measurement, after 15 min of quiet supine rest in the temperature-controlled room, as the mean over the final minute of rest; a single occasion was obtained per participant and test–retest reliability was not assessed.

### 2.7. Sample Size and Attainable Precision

At the design stage, the sample size was estimated from the correlation reported by Valdebenito et al. [45], who observed *r* = 0.74 between anthropometrically estimated fat mass [46] and MFO in individuals with low cardiometabolic risk. Applying Fisher’s z transformation for a single correlation, N = [(1.960 + 0.842)/arctanh(r)]^2^ + 3 with two-sided α = 0.05 and 1 − *β* = 0.80, returns N ≈ 12 for *r* = 0.74 and N ≈ 29 for a more conservative *r* = 0.50. That reference population, sedentary women with an at-risk body fat percentage, differs substantially from national-team athletes in age, training status, body composition and the expected magnitude of association, and it was selected at the design stage only because directly comparable athletic data were unavailable. The design-stage calculation should therefore be regarded as a weak justification, and it provides no formal support for multivariable regression, multiple testing or subgroup analysis.

For this reason, the adequacy of the sample is framed here in terms of attainable precision rather than retrospective power, which is a deterministic function of the observed *p* value and carries no additional information. With *N* = 43, a correlation of 0.50 is estimated with a confidence interval spanning approximately 0.23 to 0.70; in a subgroup of 16 participants, the same point estimate spans approximately 0.01 to 0.80. The total sample therefore supports estimation of a moderate-to-strong association with useful, though not narrow, precision, whereas subgroup-specific estimates cannot meaningfully discriminate between weak and strong associations. This consideration, rather than any post hoc power computation, is the reason that all subgroup analyses in this report are presented as exploratory and are confined to Appendix A.

### 2.8. Statistical Analysis

Distributional assumptions were assessed with the Shapiro–Wilk test, and descriptive data are presented as medians and interquartile ranges. Between-sex comparisons used the Mann–Whitney U test, with the probability of superiority reported as an effect size; *p* values within the descriptive family were adjusted with the Benjamini–Hochberg procedure to control the false discovery rate [47]. Unadjusted associations with MFO were quantified with Spearman’s rank correlation, interpreted according to conventional thresholds [48], with 95% confidence intervals obtained from 5000 bootstrap resamples and false-discovery-rate control applied across the full set of candidate variables.

The primary model was an ordinary least-squares regression of absolute MFO on absolute VO_2_peak. Sensitivity models added, one at a time and in pre-defined combinations, each skinfold-corrected girth, the uncorrected calf girth, DXA-derived FFM and fat mass, sex, stature, body mass, age, resting heart rate and the sport energy-system grouping. Two further specifications were examined: an allometric model relating the natural logarithm of MFO to the natural logarithm of absolute VO_2_peak, and a model in which MFO was scaled to FFM. The size-normalised specification, in which MFO is regressed on body-mass-normalised VO_2_peak, was fitted explicitly for comparison.

Model performance is reported as the coefficient of determination (*R*^2^), the adjusted *R*^2^, and the leave-one-out cross-validated *Q*^2^, which quantifies predictive performance in observations not used to estimate the coefficients and is therefore the informative quantity when apparent fit may be optimistic. The Akaike information criterion is reported for parsimony, and the variance inflation factor for collinearity. Regression diagnostics comprised the Breusch–Pagan test for heteroscedasticity, the Ramsey RESET test for functional form, the Durbin–Watson statistic, Shapiro–Wilk testing of residuals and Cook’s distance for influence. Because residuals departed from normality, confidence intervals for all regression coefficients were additionally obtained by bootstrapping (5000 resamples), and a robust regression with Huber weighting was fitted as a further check. Across the family of eight regional anthropometric variables tested for incremental contribution, false-discovery-rate control was applied to the corresponding *p* values.

An exploratory unsupervised analysis was also performed and is reported in full in Appendix A. Analyses were conducted in Python 3.12 (pandas, numpy, scipy, statsmodels, scikit-learn, matplotlib) with the random seed fixed at 42; the analysis script and a variable dictionary are provided as Supplementary File S1.

## 3. Results

### 3.1. Participant Characteristics

The analytic sample comprised 43 athletes (21 men, 22 women) with complete data on all study variables. Median MFO was 0.730 (0.605–0.870) g·min^−1^, median absolute VO_2_peak 3.19 (2.74–3.94) L·min^−1^ and median body-mass-normalised VO_2_peak 53.10 (45.30–60.52) mL·kg^−1^·min^−1^. Men were heavier and taller than women and had greater FFM, greater absolute VO_2_peak, larger corrected girths and higher absolute MFO; the two groups did not differ appreciably in age, adiposity, resting heart rate or MFO expressed per kilogram of FFM (Table 1).

### 3.2. Unadjusted Associations with Maximal Fat Oxidation

**Figure 2:**
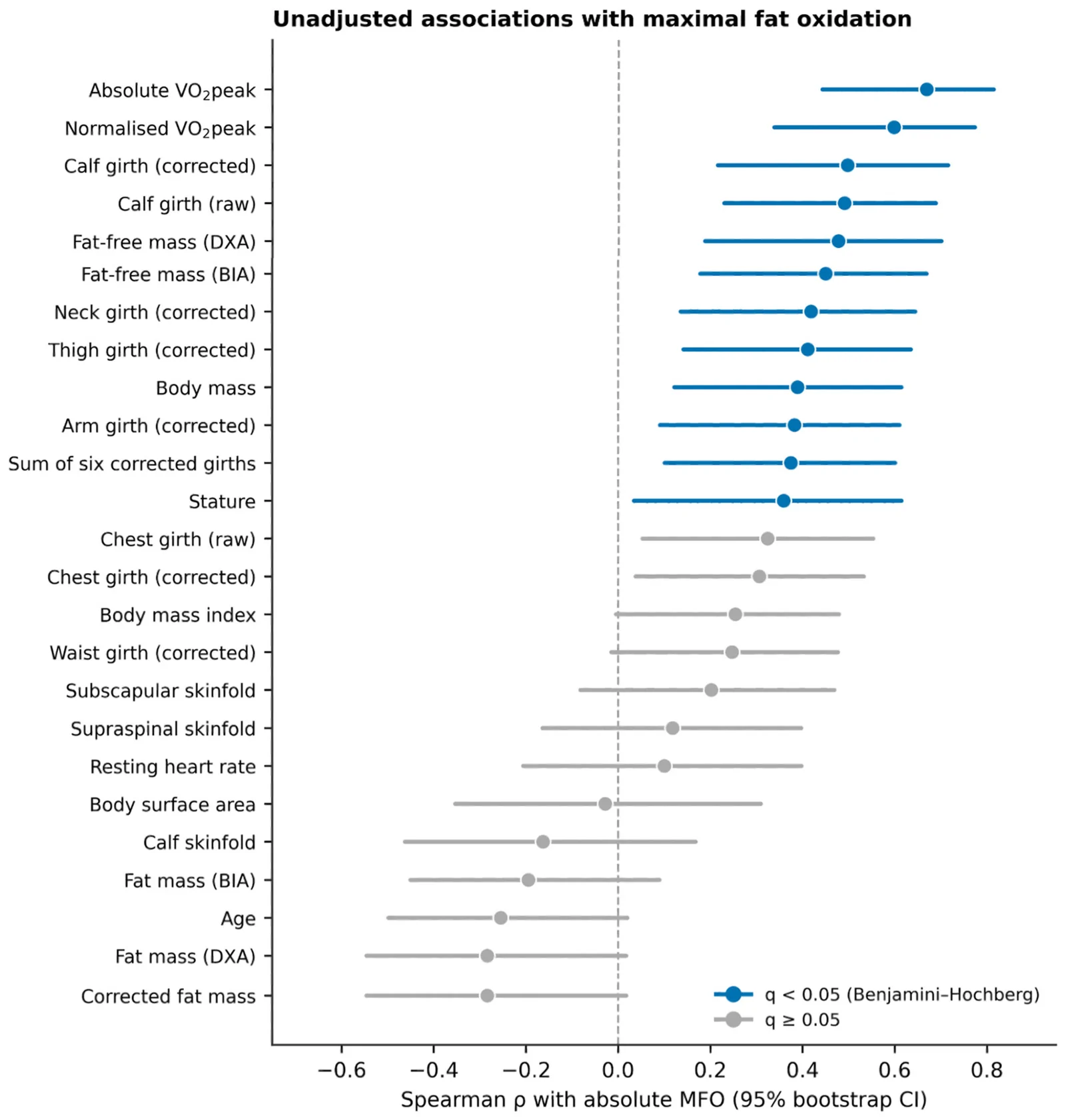
Unadjusted Spearman correlations between candidate variables and absolute maximal fat oxidation, with 95% bootstrap confidence intervals. Variables shown in colour remained significant after Benjamini–Hochberg control of the false discovery rate.

Absolute VO_2_peak showed the strongest unadjusted association with MFO (*ρ* = 0.667, 95% CI 0.44 to 0.81, *q* < 0.001), followed by the body-mass-normalised expression of the same measurement (*ρ* = 0.597, 0.34 to 0.77, *q* < 0.001). Several body-size descriptors were also associated with MFO, including uncorrected calf girth (*ρ* = 0.489), corrected calf girth (*ρ* = 0.496), FFM measured by DXA (*ρ* = 0.476) and body mass (*ρ* = 0.389). No skinfold and no measure of adiposity was associated with MFO after false-discovery-rate control (Table 2, Figure 2). The pattern is consistent with a set of mutually correlated size-related variables rather than with several independent determinants.

### 3.3. Primary Model

Absolute VO_2_peak alone explained 60.4% of the variance in absolute MFO (adjusted *R*^2^ = 0.594; *F*_1,41_ = 62.5, *p* < 0.001), with a leave-one-out cross-validated *Q*^2^ of 0.563, indicating that predictive performance was retained in observations not used for estimation. Each additional litre of oxygen consumed per minute at peak corresponded to an increase of 0.191 g·min^−1^ in MFO (95% CI 0.142 to 0.240; bootstrap CI 0.133 to 0.227). Diagnostics were satisfactory: there was no evidence of heteroscedasticity (Breusch–Pagan *p* = 0.900), of misspecified functional form (RESET *p* = 0.210) or of residual autocorrelation (Durbin–Watson = 2.01). Residuals departed modestly from normality (Shapiro–Wilk *p* = 0.009), which is why bootstrap intervals are reported throughout; a robust regression with Huber weighting yielded an essentially unchanged coefficient. Two observations exceeded the conventional Cook’s distance threshold, and leaving any single participant out changed the coefficient only marginally.

The allometric formulation gave a scaling exponent of 0.865 (95% CI 0.621 to 1.110) for the relationship between MFO and absolute VO_2_peak. The interval includes unity, so the data are compatible with an approximately proportional relationship and provide no evidence that a non-linear transformation is required.

### 3.4. Contribution of Regional Anthropometry and Body Composition

Adding corrected calf girth to the primary model increased apparent explained variance by 0.006 (*β* = +0.0092 g·min^−1^·cm^−1^, 95% CI −0.0138 to 0.0322; *p* = 0.424) and reduced cross-validated performance from *Q*^2^ = 0.563 to *Q*^2^ = 0.541, with a worse information criterion (ΔAIC = +1.3). The uncorrected calf girth behaved similarly. When each of the eight regional anthropometric variables was added in turn, none survived false-discovery-rate control; the smallest adjusted *p* value was 0.318, for corrected chest girth, whose unadjusted coefficient was negative (Table 3).

**Figure 3:**
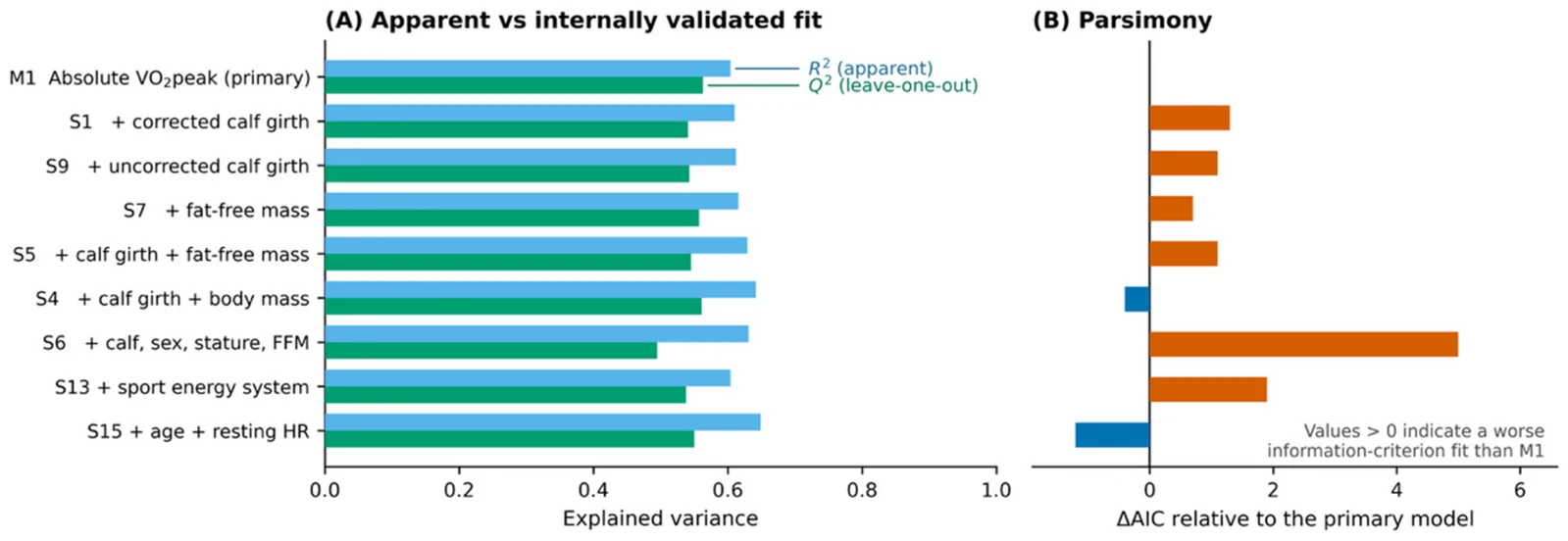
Comparison of the primary model with pre-defined sensitivity models. (**A**) Apparent explained variance (*R*^2^) alongside leave-one-out cross-validated performance (*Q*^2^). (**B**) Difference in the Akaike information criterion relative to the primary model; positive values indicate a worse information-criterion fit.

DXA-derived FFM did not contribute beyond absolute VO_2_peak (*β* = −0.0045, *p* = 0.268), and neither did fat mass, sex, stature, body mass, age, resting heart rate or the sport energy-system grouping (Table 4). In the model containing absolute VO_2_peak, corrected calf girth and FFM simultaneously, neither morphological variable approached conventional significance (*p* = 0.244 and *p* = 0.165, respectively). Age was the only additional covariate with an unadjusted *p* value below 0.05 (*β* = −0.0098 g·min^−1^·y^−1^, *p* = 0.035), a single result within a large family of sensitivity analyses that should not be interpreted as an independent finding. When MFO was scaled to FFM, the resulting outcome was unrelated to both aerobic capacity and corrected calf girth (*R*^2^ = 0.032; *Q*^2^ = −0.106), indicating that the association documented here concerns absolute oxidative flux rather than a quality intrinsic to lean tissue (Figure 3).

### 3.5. Consequences of the Size-Normalised Specification

**Figure 4:**
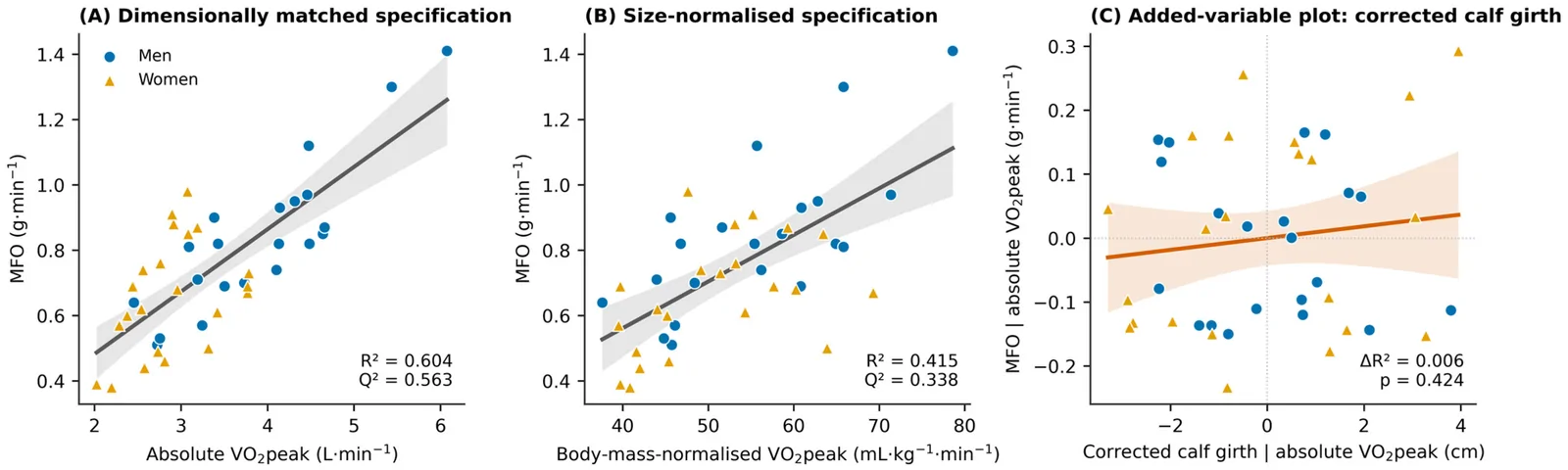
Specification of the association between aerobic capacity and maximal fat oxidation. (**A**) Absolute MFO regressed on absolute VO_2_peak (dimensionally matched). (**B**) The same outcome regressed on body-mass-normalised VO_2_peak. (**C**) Added-variable plot showing the association between corrected calf girth and MFO once absolute VO_2_peak is accounted for. Shaded areas are 95% confidence bands for the fitted line. MFO, maximal fat oxidation; VO_2_peak, peak oxygen uptake.

Replacing absolute VO_2_peak with the body-mass-normalised expression of the same measurement produced a substantially poorer model (*R*^2^ = 0.415, *Q*^2^ = 0.338, AIC = −28.4 vs. −45.2 for the primary model). In that specification, corrected calf girth appeared to contribute strongly and independently (*β* = +0.0366 g·min^−1^·cm^−1^, 95% CI 0.0170 to 0.0561; *p* < 0.001), and the two-predictor model reached *R*^2^ = 0.569, still below the fit achieved by absolute VO_2_peak alone. The apparent independent contribution of limb girth was therefore present only when body size had been divided out of the aerobic capacity term, and it disappeared entirely when the outcome and the predictor were expressed on the same dimensional scale (Figure 4A vs. Figure 4B,C).

### 3.6. Exploratory Analyses

Two sets of exploratory analyses were performed and are reported in full in the Appendix A. First, models were fitted separately in men and women. The association between absolute VO_2_peak and MFO was strong in men (*R*^2^ = 0.760, *Q*^2^ = 0.702; *β* = 0.219, 95% CI 0.160 to 0.278, *p* < 0.001) and weak and imprecisely estimated in women (*R*^2^ = 0.168, *Q*^2^ = 0.024; *β* = 0.140, 95% CI −0.005 to 0.285, *p* = 0.058). Corrected calf girth contributed in neither stratum (*p* = 0.518 in men and *p* = 0.173 in women; see Table 5). Given the sample sizes involved (*n* = 21 and *n* = 22), the confidence intervals for a correlation of this magnitude span most of the plausible range (Table 6), and this contrast should be regarded as hypothesis-generating rather than as evidence of a sex-specific mechanism.

**Figure A1:**
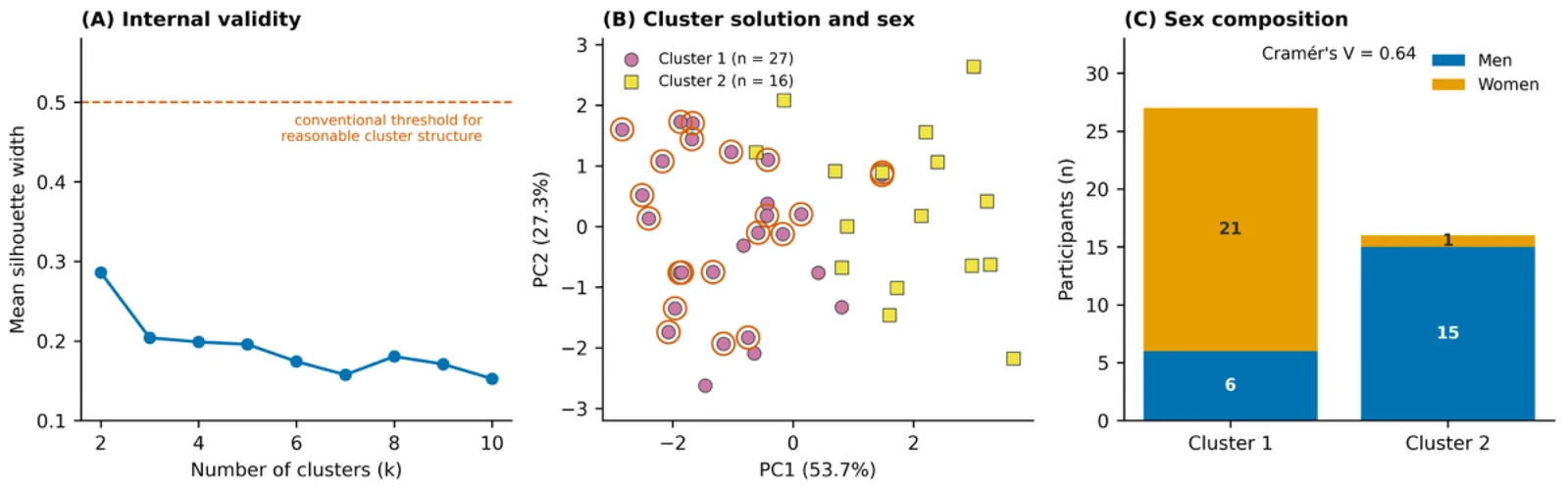
Diagnostics of the exploratory cluster analysis. (**A**) Mean silhouette width by number of clusters. (**B**) Two-cluster solution in the space of the first two principal components; open orange circles identify female participants. (**C**) Sex composition of the recovered clusters.

**Figure A2:**
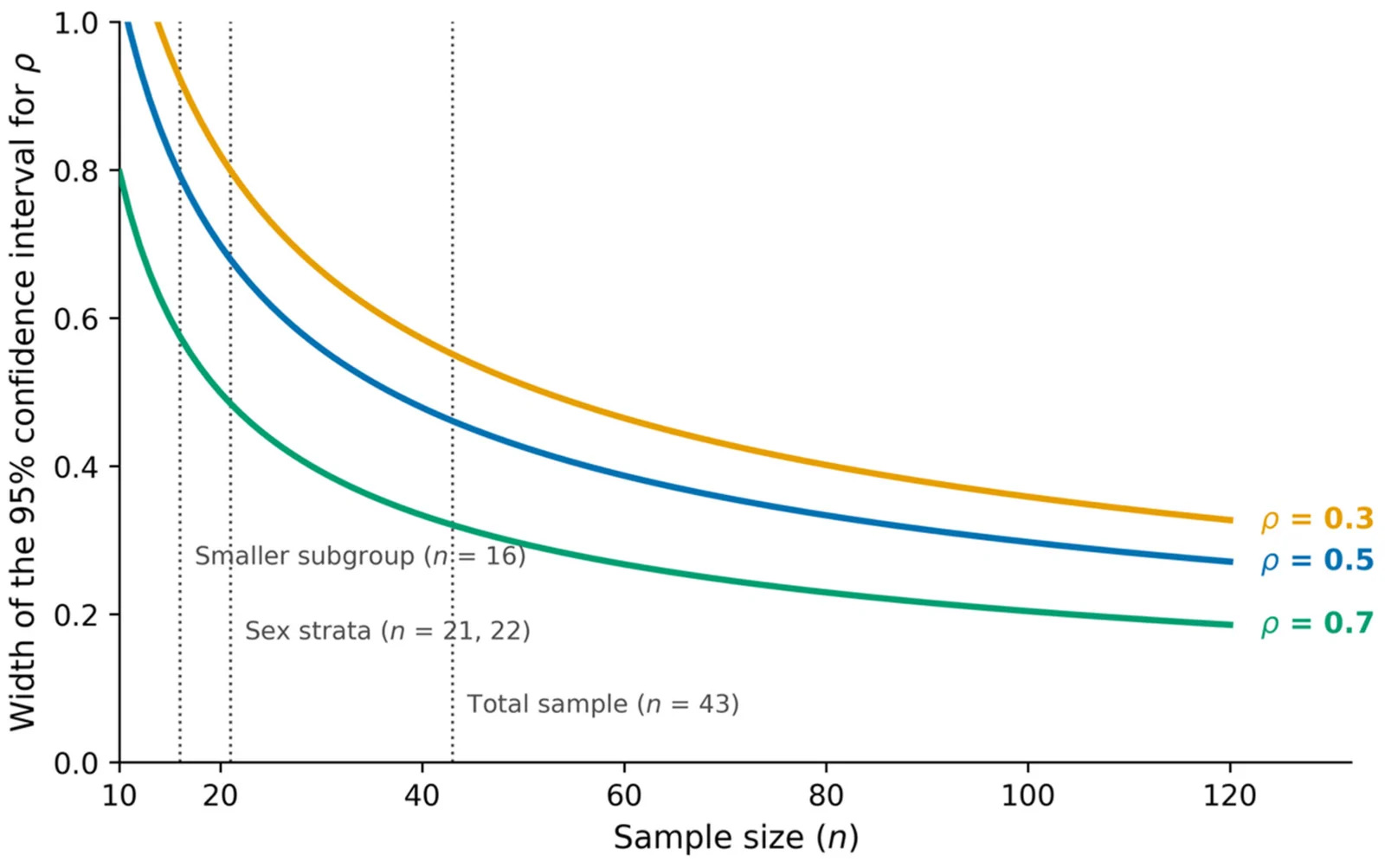
Width of the 95% confidence interval for a correlation coefficient as a function of sample size, at three values of the population correlation. Dotted lines mark the sample sizes relevant to this study.

Second, an unsupervised k-means analysis of body-composition and anthropometric variables was performed. A two-cluster solution had the highest mean silhouette width, but that value (0.286) is well below the threshold conventionally taken to indicate reasonable separation [49], and although cluster membership was stable under resampling (Jaccard 0.93 and 0.89) [50], the partition corresponded very largely to sex (Cramér’s *V* = 0.64; Fisher’s exact *p* < 0.001; one of 16 members of the second cluster was female). The clusters are therefore best understood as a sample-derived restatement of the sex difference in body size rather than as evidence of distinct morphophysiological phenotypes, and no cluster-specific regression models are reported in the main text. Full details are given in Appendix A (Table A1 and Table A2 and Figure A1 and Figure A2).

## 4. Discussion

The principal finding of this study is that absolute aerobic capacity accounted for the greater part of the between-individual variability in absolute MFO in national-team collegiate athletes, and that no anthropometric or body-composition variable examined added information beyond it. A single predictor explained 60.4% of the variance with a cross-validated *Q*^2^ of 0.563; the addition of corrected calf girth changed apparent explained variance by 0.6 percentage points (Δ*R*^2^ = 0.006), worsened out-of-sample performance and was not distinguishable from zero.

### 4.1. Specification Rather than Peripheral Morphology

The most consequential observation concerns why a limb’s girth can appear to be an independent correlate of MFO. When the same outcome was regressed on body-mass-normalised VO_2_peak, corrected calf girth was strongly and significantly associated with MFO. That association was not reproduced when aerobic capacity was expressed in absolute terms. Normalising oxygen uptake to body mass removes body-size information from the predictor while leaving it in the outcome, and limb girth, which correlates with body mass, stature and FFM, can then re-enter the model as a partial surrogate for what was removed. Tanner identified this form of spurious correlation in relation to per-weight standards [24], and the present data provide a direct empirical demonstration of it in the context of fat oxidation.

This has implications beyond the present dataset. MFO is customarily reported in grams per minute, and cardiorespiratory fitness is customarily reported per kilogram of body mass, so the mismatch described here is not unusual in this literature. Reported associations between morphological variables and MFO obtained under that specification may therefore reflect an incompletely modelled body-size term rather than a peripheral morphological determinant. This is not a claim that regional morphology is irrelevant to substrate metabolism in principle; it is a claim that the present data, analysed on a consistent dimensional scale, provide no support for such a contribution, and that the analytical choice determines the answer obtained.

### 4.2. Interpretation of the Primary Association

The magnitude of the association between absolute VO_2_peak and MFO is consistent with the literature, in which cardiorespiratory fitness is the most reproducible correlate of MFO [8,9,19]. The estimated scaling exponent, with a confidence interval including unity, indicates a relationship compatible with proportionality: athletes with a greater capacity to consume oxygen at peak also oxidise more fat per minute at their optimum submaximal intensity. This is unsurprising physiologically, since whole-body fat oxidation during exercise is constrained by mitochondrial oxidative capacity and by the delivery of oxygen and substrate to working muscle [51].

Importantly, the association concerns absolute flux. When MFO was expressed per kilogram of FFM, it was unrelated to aerobic capacity and to every morphological variable examined. The data are therefore compatible with the interpretation that a larger, fitter athlete oxidises more fat per minute chiefly because more oxidative tissue is engaged at a higher absolute metabolic rate, and not because the lean tissue of such athletes possesses a qualitatively greater oxidative capacity per unit mass. Distinguishing these possibilities would require measurements such as muscle biopsy, magnetic resonance spectroscopy or tracer methods that were not available here.

### 4.3. Corrected Girths as Surrogates

Corrected girths have proved useful for estimating whole-body quantities such as resting energy expenditure [22,23], and their appeal in applied settings is evident. The present results delimit that usefulness. Corrected girths are geometric quantities derived from a surface girth and a single skinfold; they contain no information about fibre-type composition, capillarisation, mitochondrial density or intramuscular lipid content, all of which are relevant to regional oxidative capacity [51,52]. It therefore follows that, once whole-body oxidative capacity is accounted for, a surface measurement of a single limb segment adds nothing. Practitioners seeking to characterise fat-oxidation capacity should not expect anthropometric surrogates to substitute for a metabolic measurement, and no intervention aimed at altering calf morphology can be justified on the basis of these data. Where the objective is to increase fat-oxidation capacity, the levers supported by experimental evidence remain training and dietary manipulation [53].

### 4.4. Sex and Exploratory Groupings

The association between absolute VO_2_peak and MFO was considerably stronger in men than in women. Several interpretations are possible: a narrower range of absolute VO_2_peak among the female athletes, greater residual variability related to menstrual-cycle or hormonal factors that the self-reported cycle phase cannot fully control [42], or simple sampling variation in strata of about twenty participants. The precision analysis in Table 6 shows that a correlation of 0.50 in a stratum of this size is compatible with values from approximately 0.09 to 0.77, so these strata cannot discriminate between competing explanations. The contrast is reported for transparency and as a hypothesis for adequately powered work, not as a finding.

Unsupervised clustering has been applied productively to physical performance and fitness data [54,55]. In the present dataset it did not identify structure beyond sex. The silhouette width was low, and although the partition was stable under resampling, stability is a property of the algorithm’s behaviour and not evidence that the recovered groups are biologically meaningful [49,50]. Presenting such a partition as a set of morphophysiological phenotypes would overstate what the data support, and the corresponding analyses are accordingly confined to the Appendix A.

### 4.5. Strengths and Limitations

The strengths of this study include a homogeneous, internationally competitive sample assessed under controlled laboratory conditions; complete data with no imputation; DXA as the criterion body-composition method; a full ISAK profile obtained by a single Level 2 anthropometrist with documented technical error; a single fixed, theory-informed primary model with all other analyses declared in advance as sensitivity or exploratory; internal validation by cross-validation and bootstrapping; and control of the false discovery rate within each family of tests.

Several limitations must temper interpretation. First, the design is cross-sectional, so no causal or temporal inference is available, and the results cannot indicate whether an intervention that raises aerobic capacity would raise MFO, a question that requires longitudinal designs [56]. Second, the incremental protocol advanced in 2 km·h^−1^ steps of 3 min from a starting speed of 6 km·h^−1^. In athletes of this calibre, this yields relatively few submaximal stages within the intensity range where MFO typically occurs, and MFO was determined as the highest stage-mean rate rather than from a fitted fat-oxidation curve. Both features reduce the resolution with which MFO and its associated intensity can be located, and this is the most important methodological constraint on these data; protocols with finer increments and curve-fitting procedures are preferable and are recommended for replication [1,6,20]. Third, VO_2_peak was accepted on secondary criteria without a verified plateau. Fourth, the fasting window of 6–8 h is at the shorter end of published practice and may have influenced absolute MFO values [1], and pre-test dietary intake, which is an independent determinant of MFO [14], was neither standardised nor quantified. Fifth, menstrual-cycle phase and contraceptive use were established by self-report without hormonal confirmation. Sixth, the sample comprises athletes from heterogeneous disciplines; although the energy-system grouping did not contribute to any model, the number of athletes within each discipline is too small to exclude discipline-specific effects. Seventh, with 43 participants, the estimates reported here are moderately precise for the total sample and imprecise within strata, as quantified in Table 6. Finally, these athletes were assessed in a single laboratory during the general preparation phase; generalisation to other populations, training phases, exercise modalities or protocols requires external replication.

## 5. Conclusions

In national-team collegiate athletes, absolute peak oxygen uptake accounted for the majority of the variance in absolute maximal fat oxidation, and neither skinfold-corrected girths nor fat-free mass nor any other body-size descriptor added information beyond it. An apparently independent association between corrected calf girth and MFO emerged only when aerobic capacity was expressed per kilogram of body mass, and disappeared when outcome and predictor were placed on the same dimensional scale. These findings indicate that a single, dimensionally consistent cardiorespiratory measurement is sufficient to characterise the relationship examined here, and that anthropometric surrogates of regional muscularity should not be expected to substitute for a metabolic assessment of fat-oxidation capacity. They also suggest that some previously reported morphological correlates of MFO warrant re-examination under a dimensionally consistent specification.

## Figures and Tables

**Table 1:** Participant characteristics for the total sample and by sex.

Variable	Total (*N* = 43)	Men (*n* = 21)	Women (*n* = 22)	PS	*p*	*q*
Age (y)	21.0 (19.0–24.0)	21.0 (19.0–22.0)	22.0 (19.0–27.8)	0.39	0.231	0.243
Body mass (kg)	61.90 (54.10–71.50)	70.40 (62.50–77.30)	54.65 (52.20–61.80)	0.86	<0.001	<0.001
Stretch stature (cm)	169.95 (163.00–174.82)	174.95 (172.00–179.40)	163.18 (159.72–169.32)	0.91	<0.001	<0.001
Body mass index (kg·m^−2^)	21.64 (20.08–23.87)	22.54 (21.29–24.32)	21.19 (19.97–22.50)	0.66	0.067	0.093
MFO (g·min^−1^)	0.730 (0.605–0.870)	0.820 (0.700–0.930)	0.675 (0.517–0.755)	0.73	0.010	0.017
MFO per kg FFM (mg·min^−1^·kg^−1^)	15.24 (12.77–18.48)	14.24 (12.94–16.59)	15.85 (12.25–19.86)	0.44	0.536	0.536
Absolute VO_2_peak (L·min^−1^)	3.19 (2.74–3.94)	4.10 (3.25–4.48)	2.85 (2.55–3.16)	0.84	<0.001	<0.001
Normalised VO_2_peak (mL·kg^−1^·min^−1^)	53.10 (45.30–60.52)	55.68 (46.10–62.80)	50.27 (42.51–57.05)	0.65	0.096	0.126
Resting heart rate (beats·min^−1^)	55.0 (51.0–63.0)	58.0 (52.0–63.0)	53.5 (50.5–58.5)	0.62	0.176	0.207
Fat-free mass, DXA (kg)	48.47 (40.78–54.31)	53.80 (50.39–62.17)	40.88 (38.18–46.49)	0.93	<0.001	<0.001
Fat mass, DXA (kg)	12.84 (9.48–14.70)	12.00 (9.06–14.37)	13.72 (10.89–15.68)	0.39	0.220	0.243
Fat-free mass, BIA (kg)	52.60 (44.20–61.00)	60.60 (53.30–64.80)	45.10 (41.02–51.65)	0.93	<0.001	<0.001
Fat mass, BIA (kg)	11.10 (7.90–12.65)	10.70 (6.60–12.40)	12.15 (9.62–12.67)	0.38	0.177	0.207
Calf girth, uncorrected (cm)	34.45 (33.38–36.15)	35.50 (34.20–37.00)	33.40 (32.12–35.49)	0.70	0.024	0.037
Corrected calf girth (cm)	32.28 (30.84–34.13)	33.18 (31.82–34.76)	31.29 (30.13–33.15)	0.71	0.018	0.029
Corrected thigh girth (cm)	46.37 (44.10–49.39)	48.15 (45.94–52.07)	44.61 (43.08–46.45)	0.79	0.001	0.002
Corrected arm girth (cm)	26.00 (23.33–29.53)	29.37 (26.62–31.64)	23.33 (22.06–25.24)	0.92	<0.001	<0.001
Corrected neck girth (cm)	31.16 (28.84–34.34)	34.57 (32.62–35.93)	28.84 (27.94–30.22)	0.94	<0.001	<0.001
Corrected chest girth (cm)	86.32 (80.89–92.64)	92.81 (87.68–98.96)	81.15 (78.94–85.20)	0.89	<0.001	<0.001
Corrected waist girth (cm)	69.38 (65.79–75.46)	75.52 (71.18–77.07)	66.08 (65.29–67.48)	0.87	<0.001	<0.001
Sum of six corrected girths (cm)	290.93 (273.29–314.23)	315.52 (302.21–325.89)	275.09 (268.48–286.61)	0.90	<0.001	<0.001

Note: Data are median (interquartile range). PS, probability of superiority (probability that a randomly chosen man exceeds a randomly chosen woman); *p*, Mann–Whitney U test; *q*, *p* value after Benjamini–Hochberg control of the false discovery rate within this table. BIA, bioelectrical impedance analysis; DXA, dual-energy X-ray absorptiometry; FFM, fat-free mass; MFO, maximal fat oxidation; VO_2_peak, peak oxygen uptake.

**Table 2:** Unadjusted Spearman correlations with absolute maximal fat oxidation (*N* = 43).

Variable	*ρ*	95% CI	*p*	*q*
Absolute VO_2_peak (L·min^−1^)	0.667	0.44 to 0.81	<0.001	<0.001
Normalised VO_2_peak (mL·kg^−1^·min^−1^)	0.597	0.34 to 0.77	<0.001	<0.001
Corrected calf girth	0.496	0.21 to 0.71	<0.001	0.006
Calf girth (uncorrected)	0.489	0.23 to 0.69	<0.001	0.006
Fat-free mass (DXA)	0.476	0.19 to 0.70	0.001	0.007
Fat-free mass (BIA)	0.450	0.18 to 0.67	0.002	0.009
Corrected neck girth	0.418	0.14 to 0.64	0.005	0.018
Corrected thigh girth	0.411	0.14 to 0.63	0.006	0.018
Body mass	0.389	0.12 to 0.61	0.010	0.027
Corrected arm girth	0.383	0.09 to 0.61	0.011	0.028
Sum of six corrected girths	0.375	0.10 to 0.60	0.013	0.030
Stretch stature	0.359	0.03 to 0.61	0.018	0.037
Chest girth (uncorrected)	0.324	0.05 to 0.55	0.034	0.065
Corrected chest girth	0.307	0.04 to 0.53	0.046	0.082
Fat mass (DXA)	−0.284	−0.55 to 0.02	0.065	0.097
Age	−0.254	−0.50 to 0.02	0.100	0.135
Body mass index	0.254	−0.00 to 0.48	0.100	0.135
Corrected waist girth	0.247	−0.02 to 0.48	0.110	0.142
Subscapular skinfold	0.202	−0.08 to 0.47	0.193	0.237
Fat mass (BIA)	−0.195	−0.45 to 0.09	0.211	0.247
Medial calf skinfold	−0.163	−0.46 to 0.17	0.297	0.334
Supraspinale skinfold	0.118	−0.16 to 0.40	0.450	0.486
Resting heart rate	0.100	−0.21 to 0.40	0.522	0.542

Note: Confidence intervals were obtained from 5000 bootstrap resamples. *q*, *p* value after Benjamini–Hochberg control of the false discovery rate across all variables in the table. BIA, bioelectrical impedance analysis; DXA, dual-energy X-ray absorptiometry.

**Table 3:** Incremental contribution of regional anthropometric variables added individually to the primary model.

Variable Added	*β* (g·min^−1^ per Unit)	95% CI	Δ*R*^2^	*Q* ^2^	*p*	*q*
Corrected calf girth	+0.0092	−0.0138 to 0.0322	0.006	0.541	0.424	0.489
Corrected thigh girth	−0.0047	−0.0204 to 0.0109	0.004	0.529	0.546	0.572
Corrected arm girth	−0.0130	−0.0289 to 0.0029	0.025	0.576	0.106	0.318
Corrected neck girth	−0.0130	−0.0308 to 0.0048	0.020	0.568	0.148	0.366
Corrected chest girth	−0.0073	−0.0141 to −0.0005	0.041	0.599	0.037	0.318
Corrected waist girth	−0.0073	−0.0154 to 0.0008	0.030	0.583	0.077	0.318
Sum of six corrected girths	−0.0021	−0.0046 to 0.0003	0.028	0.580	0.087	0.318
Calf girth (uncorrected)	+0.0104	−0.0124 to 0.0331	0.008	0.543	0.362	0.452

Note: Each row represents a separate model containing absolute VO_2_peak and the variable indicated. Δ*R*^2^, change in explained variance relative to the primary model (*R*^2^ = 0.604, *Q*^2^ = 0.563); *q*, *p* value after Benjamini–Hochberg control of the false discovery rate across the eight variables in this family.

**Table 4:** Primary and sensitivity models for absolute maximal fat oxidation (*n* = 43).

Model	*k*	*R* ^2^	Adj. *R*^2^	*Q* ^2^	AIC	ΔAIC	Max VIF
M1. Absolute VO_2_peak (primary)	1	0.604	0.594	0.563	−45.2	+0.0	1.00
S1. M1 + corrected calf girth	2	0.610	0.591	0.541	−43.9	+1.3	1.64
S9. M1 + uncorrected calf girth	2	0.612	0.593	0.543	−44.1	+1.1	1.40
S7. M1 + fat-free mass	2	0.616	0.597	0.557	−44.5	+0.7	3.35
S5. M1 + corrected calf girth + fat-free mass	3	0.629	0.601	0.545	−44.0	+1.1	3.64
S4. M1 + corrected calf girth + body mass	3	0.642	0.615	0.561	−45.5	−0.4	2.28
S3. M1 + corrected calf girth + stature	3	0.615	0.585	0.523	−42.3	+2.8	2.11
S2. M1 + corrected calf girth + sex	3	0.613	0.583	0.523	−42.1	+3.0	2.14
S6. M1 + corrected calf girth + sex + stature + fat-free mass	5	0.631	0.581	0.495	−40.2	+5.0	6.95
S13. M1 + sport energy system	2	0.604	0.585	0.538	−43.2	+1.9	1.53
S15. M1 + age + resting heart rate	3	0.649	0.622	0.550	−46.4	−1.2	1.03
A1. Allometric log–log model	1	0.555	0.544	0.515	−15.4	+29.8	1.00
S11. Outcome scaled to fat-free mass + corrected calf girth	2	0.032	−0.017	−0.106	241.3	+286.5	1.64

Abbreviations: *k*, number of predictors; *Q*^2^, leave-one-out cross-validated coefficient of determination; AIC, Akaike information criterion; ΔAIC, difference relative to the primary model, with positive values indicating a worse fit; VIF, variance inflation factor. All models are ordinary least squares with absolute maximal fat oxidation as the outcome, except A1, in which the outcome is log-transformed, and S11, in which the outcome is scaled to fat-free mass. Because the Akaike information criterion depends on the scale of the outcome, the AIC and ΔAIC values for A1 and S11 are not comparable with those of the remaining models and are shown for completeness only.

**Table 5:** Sex-stratified estimates for the primary model (exploratory).

Stratum	*β* (VO_2_peak)	95% CI	*R* ^2^	*Q* ^2^	*p*	*β* (Calf Girth)	*p* (Calf Girth)
Men (*n* = 21)	0.2192	0.1601 to 0.2783	0.760	0.702	<0.001	−0.0105	0.518
Women (*n* = 22)	0.1397	−0.0051 to 0.2845	0.168	0.024	0.058	+0.0226	0.173

Abbreviations: *β* (VO_2_peak), change in maximal fat oxidation (g·min^−1^) per litre per minute of absolute peak oxygen uptake. The final two columns are from the model additionally containing corrected calf girth. These strata are underpowered for the estimation of within-sex associations and are reported as exploratory.

**Table 6:** Attainable precision: width of the 95% confidence interval for a correlation coefficient at the sample sizes relevant to this study.

Sample Size	*ρ* = 0.30	*ρ* = 0.50	*ρ* = 0.70
43	−0.00 to 0.55 (width 0.55)	0.23 to 0.70 (width 0.46)	0.51 to 0.83 (width 0.32)
27	−0.09 to 0.61 (width 0.70)	0.15 to 0.74 (width 0.59)	0.44 to 0.85 (width 0.42)
22	−0.14 to 0.64 (width 0.78)	0.10 to 0.76 (width 0.66)	0.39 to 0.87 (width 0.47)
21	−0.15 to 0.65 (width 0.80)	0.09 to 0.77 (width 0.68)	0.38 to 0.87 (width 0.48)
16	−0.23 to 0.69 (width 0.92)	0.01 to 0.80 (width 0.79)	0.31 to 0.89 (width 0.57)

Note: Intervals were derived from Fisher’s z transformation. The rows correspond to the total sample (*N* = 43), the larger exploratory subgroup (*n* = 27), the two sex strata (*n* = 22 and *n* = 21) and the smaller exploratory subgroup (*n* = 16).

## Data Availability

The de-identified dataset supporting the conclusions of this article, together with the analysis scripts and a variable dictionary, will be made available by the corresponding author on reasonable request from qualified researchers for non-commercial academic purposes, subject to a data-use agreement. Open deposition is restricted because the participants are members of national teams and the combination of sport discipline, sex, age and anthropometric profile would render individuals re-identifiable; the consent obtained did not cover unrestricted public release. The institutional custodian of the dataset is the Nutritional Assessment and Nutritional Care Laboratory (LECEN), Tonala University Center, University of Guadalajara. Analysis code and session information are provided without restriction as Supplementary Material and correspond to the version used to generate every result reported in this manuscript.

## Supplementary Materials

The following supporting information can be downloaded at: https://www.mdpi.com/article/10.3390/jfmk11030349/s1. File S1, analysis script, variable dictionary and session information for the software environment used.

## Abbreviations

The following abbreviations are used in this manuscript:

AIC	Akaike information criterion
BIA	Bioelectrical impedance analysis
CI	Confidence interval
DXA	Dual-energy X-ray absorptiometry
FDR	False discovery rate
FFM	Fat-free mass
ISAK	International Society for the Advancement of Kinanthropometry
MFO	Maximal fat oxidation
PS	Probability of superiority
*Q*^2^	Cross-validated coefficient of determination
USG	Urine specific gravity
VIF	Variance inflation factor
VO_2_peak	Peak oxygen uptake

## Appendix A

**Table A1:** Descriptive characteristics by cluster (exploratory).

Variable	Cluster 1 (*n* = 27)	Cluster 2 (*n* = 16)	*PS*	*p*
Age (y)	21.0 (18.5–24.0)	21.0 (19.0–22.5)	0.49	0.919
Body mass (kg)	57.60 (52.30–61.70)	73.90 (70.08–79.50)	0.00	<0.001
Stretch stature (cm)	164.90 (160.45–170.82)	175.45 (172.30–179.40)	0.14	<0.001
Body mass index (kg·m^−2^)	20.43 (19.35–21.74)	24.10 (22.40–25.25)	0.11	<0.001
MFO (g·min^−1^)	0.680 (0.520–0.815)	0.835 (0.725–0.935)	0.24	0.005
MFO per kg FFM (mg·min^−1^·kg^−1^)	15.51 (11.86–19.87)	13.96 (12.97–16.54)	0.55	0.572
Absolute VO_2_peak (L·min^−1^)	2.90 (2.57–3.25)	4.14 (3.64–4.52)	0.12	<0.001
Normalised VO_2_peak (mL·kg^−1^·min^−1^)	53.10 (44.42–59.77)	53.64 (46.61–61.38)	0.42	0.393
Resting heart rate (beats·min^−1^)	54.0 (50.0–59.5)	59.0 (52.8–63.0)	0.37	0.174
Fat-free mass, DXA (kg)	42.56 (38.57–48.06)	57.12 (53.43–64.30)	0.03	<0.001
Fat mass, DXA (kg)	12.51 (9.48–14.32)	13.70 (9.41–16.31)	0.42	0.414
Fat-free mass, BIA (kg)	45.70 (41.85–52.55)	62.75 (60.12–66.62)	0.01	<0.001
Fat mass, BIA (kg)	11.00 (8.15–12.55)	11.40 (7.82–14.18)	0.43	0.482
Calf girth, uncorrected (cm)	33.40 (31.80–34.95)	36.55 (34.56–37.16)	0.15	<0.001
Corrected calf girth (cm)	31.24 (30.13–32.93)	34.13 (32.44–34.89)	0.19	<0.001
Corrected thigh girth (cm)	44.74 (43.11–46.42)	49.78 (48.01–53.12)	0.09	<0.001
Corrected arm girth (cm)	24.04 (22.49–25.72)	30.33 (29.17–31.94)	0.02	<0.001
Corrected neck girth (cm)	29.26 (28.41–30.82)	35.25 (33.86–36.14)	0.04	<0.001
Corrected chest girth (cm)	81.39 (79.19–85.06)	93.98 (92.26–99.46)	0.00	<0.001
Corrected waist girth (cm)	66.29 (65.01–68.48)	75.96 (74.91–77.78)	0.02	<0.001
Sum of six corrected girths (cm)	276.89 (269.01–288.05)	320.89 (312.07–330.41)	0.00	<0.001

Note: Data are median (interquartile range). PS, probability of superiority. Cluster 1 comprised 6 men and 21 women; cluster 2 comprised 15 men and 1 woman. These groupings are sample-derived and are reported for transparency only.

**Table A2:** Internal validity and stability of the exploratory cluster solution.

Metric	Value
Mean silhouette width (*k* = 2)	0.286
Mean silhouette width (*k* = 3)	0.204
Bootstrap Jaccard stability, cluster 1	0.93
Bootstrap Jaccard stability, cluster 2	0.89
Cramér’s V with sex	0.64
Fisher’s exact test against sex	*p* < 0.001
Adjusted Rand index, reduced variable set	0.58

Note: Silhouette widths below approximately 0.50 indicate weak separation. Jaccard values were obtained from 1000 bootstrap resamples. The association with sex indicates that the partition largely reproduces the sex difference in body size.
