# Supplementary material for: Absolute Aerobic Capacity Predominates over Body Composition and Regional Anthropometry in Explaining Maximal Fat Oxidation in National-Team Collegiate Athletes: A Cross-Sectional Study
Source: J Funct Morphol Kinesiol. 2026 Sep 3;11(3):349. doi: 10.3390/jfmk11030349 (PMC13608663; doi:10.3390/jfmk11030349)
Supplement: Supplementary file 1 [file jfmk-11-00349-s001.zip › jfmk-4469372-supplementary.pdf]

Supplementary File S1

Analysis script, variable dictionary and session information for the software environment used.

Absolute Aerobic Capacity Predominates Over Body Composition and Regional Anthropometry in Explaining Maximal Fat Oxidation in National-Team Collegiate Athletes: A Cross-Sectional Study

---

1. Software environment

The versions below were written by the script itself at run time and are the versions used to generate the results reported in the article.

Component	Version
Python	3.12.3
Platform	Linux-6.18.44-fc-v21-x86_64-with-glibc2.39
numpy	2.4.4
pandas	3.0.2
scipy	1.17.1
statsmodels	0.14.6
scikit-learn	1.8.0
matplotlib	3.10.8
Random seed	42
Bootstrap resamples	5000 (correlations, coefficients); 1000 (cluster stability)

2. Variable dictionary

The analytic dataset contains one row per participant (n = 43) and 31 columns, with no missing values. Entries numbered 1-31 are columns of the workbook; entries D1-D5 are variables constructed inside the script and are listed so that every quantity entering a model is defined. "Derived" in the Type column means that the variable is an arithmetic function of other variables; each such relation was verified numerically and the result is reported in Section 5.

#	Column in workbook	Name in script	Label used in the article	Unit	Type	Definition, derivation and provenance	Role in the analysis
1	MFO (g/m)	mfo	Maximal fat oxidation (MFO)	g/min	Continuous	Highest rate of fat oxidation attained during the maximal incremental treadmill test. Breath-by-breath indirect calorimetry (PNOE, ENDO Medical, serial PNOE 2116-393) with a 3-breath moving-average filter; substrate oxidation computed from the mean of the final 60 s of each 3-min stage using stoichiometric equations for non-protein respiratory quotient. Despite the header abbreviation "g/m", the unit is grams per minute.	Primary outcome of every model (Sections 3.2-3.6)
2	VO2peak_ABS(L/min)	vo2_abs	Absolute VO2peak	L/min	Continuous	Peak oxygen uptake in absolute terms, from the same ergospirometry session. Accepted after volitional exhaustion when at least two secondary criteria were met (RER >= 1.10; >= 95% of age-predicted maximal heart rate; RPE >= 9 on the Borg 1-10 scale). No oxygen-uptake plateau criterion was applied, hence "peak" rather than "maximum".	Sole predictor in the primary model M1; component of every sensitivity model
3	VO2peak	vo2_rel	Normalised VO2peak	mL/kg/min	Continuous	The same measurement expressed per kilogram of body mass. Verified to equal absolute VO2peak / body mass x 1000 for every record.	Predictor in the size-normalised specification (Section 3.5), used to demonstrate the consequences of the dimensional mismatch
4	Age	age	Age	years	Continuous	Age at the date of assessment. Range 16-39 years (median 21).	Covariate in sensitivity models S15; descriptive (Tables 1, A1); candidate correlate (Table 2)
5	Sex	sex	Sex	-	Categorical	1 = men (n = 21); 2 = women (n = 22). Recoded in the script to the indicator variable `male` (1 = men, 0 = women) for regression.	Stratifying variable (Table 5); covariate in models S2 and S6; grouping variable in Table 1
6	Body.Mass	mass	Body mass	kg	Continuous	Digital scale accurate to 100 g (Seca 874, Hamburg, Germany), measured in tight-fitting clothing without metallic objects.	Covariate in model S4; candidate correlate (Table 2)
7	Stature	stature	Stretch stature	cm	Continuous	Stretch stature obtained according to the ISAK protocol by a single Level 2 certified anthropometrist.	Covariate in models S3 and S6; candidate correlate (Table 2)
8	IMC	bmi	Body mass index	kg/m2	Derived	Body mass divided by squared stature in metres. Verified to reproduce exactly from columns 6 and 7. ("IMC" is the Spanish acronym.)	Descriptive (Tables 1, A1); candidate correlate (Table 2)
9	BSA	bsa	Body surface area	m2	Derived	Body surface area. The values in this column match the Du Bois equation once each set is sorted independently (largest gap 0.0005 m2), but they do NOT match record by record, indicating that the column is not aligned to the correct participants. See the data-integrity note in Section 5 of this file; the script reports both the supplied and the recomputed value.	Candidate correlate only (Table 2). Not a predictor in any model, so no model result is affected
10	FFM.DXA	ffm_dxa	Fat-free mass, DXA	kg	Continuous	Fat-free mass by dual-energy X-ray absorptiometry (Lunar iDXA, General Electric, Madison, WI, USA), enCORE platform version 18, standard scan mode, supine, with daily calibration.	Covariate in models S5, S6, S7; denominator of the FFM-scaled outcome (model S11); candidate correlate (Table 2)
11	FM.DXA	fm_dxa	Fat mass, DXA	kg	Continuous	Fat mass from the same DXA acquisition.	Sensitivity covariate; candidate correlate (Table 2)
12	FFAT	ffat	Fat-free adipose tissue	kg	Derived	Non-lipid component of adipose tissue, computed as a fixed fraction (0.1765) of DXA fat mass; verified to reproduce from column 11 for every record. Because it is a constant multiple of fat mass, its rank correlation with any variable is identical to that of fat mass.	Candidate correlate (Table 2)
13	FM.Correct.DXa	fm_corr	Corrected fat mass	kg	Derived	Adipose-tissue mass, computed as DXA fat mass plus fat-free adipose tissue (columns 11 + 12); verified exactly for every record. Also a constant multiple of fat mass, hence the identical rank correlation.	Candidate correlate (Table 2)
14	Muss.index	muscle_index	Muscle index	kg/m2	Derived	Fat-free mass index: DXA fat-free mass divided by squared stature in metres. Verified to reproduce exactly from columns 10 and 7.	Candidate correlate (Table 2)
15	FFM.BIA	ffm_bia	Fat-free mass, BIA	kg	Continuous	Fat-free mass by multifrequency bioelectrical impedance analysis (InBody 720, InBody Co, Cerritos, CA, USA), exported with LookinBody version 3.0; derived as body mass minus fat mass. Standardised by a 6-8 h overnight fast and bladder voiding immediately before measurement.	Descriptive (Tables 1, A1); candidate correlate (Table 2); entered into the exploratory cluster analysis

#	Column in workbook	Name in script	Label used in the article	Unit	Type	Definition, derivation and provenance	Role in the analysis
16	FM.BIA	fm_bia	Fat mass, BIA	kg	Continuous	Fat mass from the same BIA acquisition.	Descriptive (Tables 1, A1); candidate correlate (Table 2); entered into the exploratory cluster analysis
17	Calf.SF	sf_calf	Medial calf skinfold	mm	Continuous	Medial calf skinfold, ISAK protocol, measured in duplicate (third measurement and median retained when duplicates differed by more than 5%). Used to compute the corrected calf girth.	Candidate correlate (Table 2); input to column 27
18	Subscapular.SF	sf_sub	Subscapular skinfold	mm	Continuous	Subscapular skinfold, ISAK protocol, same duplicate procedure. Used to compute the corrected chest girth.	Candidate correlate (Table 2); input to column 24
19	Supraspinal.SF	sf_supra	Supraspinale skinfold	mm	Continuous	Supraspinale skinfold, ISAK protocol, same duplicate procedure. Used to compute the corrected waist girth.	Candidate correlate (Table 2); input to column 25
20	Calf.G	calf_g	Calf girth (uncorrected)	cm	Continuous	Maximal medial calf girth, ISAK protocol, before subtraction of the corresponding skinfold.	Covariate in model S9; incremental-contribution family (Table 4); candidate correlate (Table 2)
21	Torax.G	chest_g	Chest girth (uncorrected)	cm	Continuous	Mesosternal chest girth, ISAK protocol, before subtraction of the corresponding skinfold. ("Torax" is the Spanish term; the workbook header carries an accent.)	Candidate correlate (Table 2)
22	Neck.CG	neck_cg	Corrected neck girth	cm	Derived	Neck girth minus pi x (submandibular skinfold in cm), following the corrected-girth convention of the ISAK-based protocol used by this laboratory. The submandibular skinfold itself is not included in the deposited dataset.	Incremental-contribution family (Table 4); candidate correlate (Table 2); entered into the exploratory cluster analysis
23	Arm.CG	arm_cg	Corrected arm girth	cm	Derived	Relaxed arm girth minus pi x (triceps skinfold in cm). The triceps skinfold itself is not included in the deposited dataset.	Incremental-contribution family (Table 4); candidate correlate (Table 2); entered into the exploratory cluster analysis
24	Chest.CG	chest_cg	Corrected chest girth	cm	Derived	Mesosternal chest girth minus pi x (subscapular skinfold in cm), i.e. column 21 minus pi x column 18 / 10. Reproduces exactly for 42 of 43 records; one record disagrees by 2.86 cm and should be rechecked (see the data-integrity table).	Incremental-contribution family (Table 4); candidate correlate (Table 2); entered into the exploratory cluster analysis
25	Waist.CG	waist_cg	Corrected waist girth	cm	Derived	Minimal waist girth minus pi x (supraspinale skinfold in cm).	Incremental-contribution family (Table 4); candidate correlate (Table 2); entered into the exploratory cluster analysis
26	Thigh.CG	thigh_cg	Corrected thigh girth	cm	Derived	Mid-thigh girth minus pi x (anterior thigh skinfold in cm). The anterior thigh skinfold itself is not included in the deposited dataset.	Incremental-contribution family (Table 4); candidate correlate (Table 2); entered into the exploratory cluster analysis
27	Calf.CG	calf_cg	Corrected calf girth	cm	Derived	Maximal calf girth minus pi x (medial calf skinfold in cm), i.e. column 20 minus pi x column 17 / 10. Verified to reproduce exactly for every record.	The a-priori variable of interest for the secondary aim: covariate in models S1-S6 and S11, in the size-normalised specification, and in both sex strata; candidate correlate (Table 2)
28	X6CG	sum6cg	Sum of six corrected girths	cm	Derived	Sum of columns 22-27. Reproduces to within 0.06 cm for 41 of 43 records; one record differs by 0.71 cm and should be rechecked (see the data-integrity table).	Incremental-contribution family (Table 4); candidate correlate (Table 2)
29	RHR	rhr	Resting heart rate	beats/min	Continuous	Resting heart rate recorded during the resting clinical screening performed by the laboratory medical staff before the maximal test.	Covariate in model S15; candidate correlate (Table 2); entered into the exploratory cluster analysis
30	sport_energy_system	energy_system	Predominant energy system of the sport	-	Categorical	Descriptive grouping of the athlete's sport by predominant energy system: 1 = oxidative (n = 14; triathlon, sailing, 5000 m running, mountain biking); 2 = glycolytic (n = 18; 400-800 m track events, 400 m hurdles, Brazilian jiu-jitsu, wushu, taekwondo, karate); 3 = phosphagen (n = 4; weightlifting, 100-200 m sprints); 4 = intermittent (n = 7; soccer, volleyball, flag football, basketball). Entered in model S13 as the binary contrast oxidative (1) versus all other sports (2-4), which reproduces the published model statistics for S13.	Covariate in sensitivity model S13
31	Cluster	cluster_published	Cluster membership (exploratory)	-	Categorical	Membership in the two-cluster k-means solution reported in the Appendix: 1 = larger cluster (n = 27, comprising 6 men and 21 women); 2 = smaller cluster (n = 16, comprising 15 men and 1 woman). Stored so that the published partition can be inspected without re-running the clustering; the script recomputes it independently and confirms exact agreement (adjusted Rand index = 1.00).	Grouping variable for Tables A1-A2 and Figure A1 (exploratory only)
D1	(derived in script)	mfo_per_ffm	MFO per kg fat-free mass	mg/min/kg	Derived	MFO divided by DXA fat-free mass, multiplied by 1000. Computed inside the script; not a column of the workbook.	Outcome of sensitivity model S11 and of the corresponding row of Tables 1 and A1
D2	(derived in script)	ln_mfo	ln(MFO)	ln(g/min)	Derived	Natural logarithm of MFO.	Outcome of the allometric model A1
D3	(derived in script)	ln_vo2_abs	ln(absolute VO2peak)	ln(L/min)	Derived	Natural logarithm of absolute VO2peak.	Predictor in the allometric model A1; its coefficient is the scaling exponent

#	Column in workbook	Name in script	Label used in the article	Unit	Type	Definition, derivation and provenance	Role in the analysis
D4	(derived in script)	oxidative_sport	Oxidative sport indicator	-	Derived	1 if the predominant energy system of the sport is oxidative (column 30 equals 1), 0 otherwise.	Covariate in sensitivity model S13
D5	(derived in script)	male	Male sex indicator	-	Derived	1 for men, 0 for women (recoded from column 5).	Covariate in models S2 and S6

3. Analysis script

Complete listing of S1_MFO_analysis.py (1389 lines). The runnable file is distributed alongside this document.

```
#!/usr/bin/env python3
# -*- coding: utf-8 -*-
"""
================================================================================
SUPPLEMENTARY FILE S1 - ANALYSIS SCRIPT
================================================================================

Manuscript
    Absolute Aerobic Capacity Predominates Over Body Composition and Regional
    Anthropometry in Explaining Maximal Fat Oxidation in National-Team
    Collegiate Athletes: A Cross-Sectional Study

Purpose
    Complete, self-contained reproduction of every quantity reported in the
    main text (Tables 1-6, Figures 2-4) and in the Appendix (Tables A1-A2,
    Figures A1-A2), from the de-identified analytic dataset.

Input
    Dataset_MFO.xlsx   (n = 43 rows, 31 columns; one row per participant,
                        no missing values). See the variable dictionary in
                        Supplementary File S1 for the definition, unit and
                        provenance of every column.

Usage
    python S1_MFO_analysis.py --data Dataset_MFO.xlsx --outdir outputs

Outputs (written to --outdir)
    data_integrity_checks.csv
    table1_participant_characteristics.csv
    table2_unadjusted_correlations.csv
    table3_models.csv
    table4_incremental_anthropometry.csv
    table5_sex_stratified.csv
    table6_attainable_precision.csv
    tableA1_cluster_descriptives.csv
    tableA2_cluster_validity.csv
    figure2_correlations.png
    figure3_specification.png
    figure4_model_comparison.png
    figureA1_cluster_diagnostics.png
    figureA2_attainable_precision.png
    primary_model_diagnostics.txt
    reproduction_check.csv
    session_info.txt
    analysis_log.txt

Reproducibility
    Random seed fixed at 42 for every stochastic procedure (bootstrap
    resampling, k-means initialisation, cluster-stability resampling).
    No imputation is performed; the analytic dataset is complete.
    No automated variable-selection procedure is used at any stage.

    NOTE ON BOOTSTRAP INTERVALS. Percentile bootstrap limits depend on the
    exact pseudo-random stream of the NumPy build in use. Re-running this
    script reproduces the published limits to within approximately +/-0.02
    on the correlation scale. Point estimates, model statistics, p values
    and cross-validated statistics are deterministic and reproduce exactly.

Software environment
    Python 3.12; pandas, numpy, scipy, statsmodels, scikit-learn, matplotlib,
    openpyxl. Exact versions are written to session_info.txt at run time.

License / data availability
    The de-identified dataset supporting this script is available from the
    corresponding author on reasonable request, subject to a data-use
    agreement (see the Data Availability Statement of the main article).
================================================================================
"""

from __future__ import annotations

import argparse
import platform
import sys
import warnings
from pathlib import Path

import matplotlib
matplotlib.use("Agg")
import matplotlib.pyplot as plt
import numpy as np
import pandas as pd
import scipy
import sklearn
import statsmodels
import statsmodels.api as sm
from scipy import stats
from sklearn.cluster import KMeans
from sklearn.decomposition import PCA
from sklearn.metrics import adjusted_rand_score, silhouette_score
from sklearn.preprocessing import StandardScaler
from statsmodels.stats.diagnostic import het_breuschpagan, linear_reset
from statsmodels.stats.multitest import multipletests
from statsmodels.stats.outliers_influence import variance_inflation_factor
from statsmodels.stats.stattools import durbin_watson

warnings.filterwarnings("ignore", category=FutureWarning)
```

```python
# ============================================================================
# 0. CONFIGURATION
# ============================================================================

SEED = 42
N_BOOT = 5000          # bootstrap resamples for correlations and coefficients
N_BOOT_STABILITY = 1000  # bootstrap resamples for cluster stability (Jaccard)
DPI = 600

# Okabe-Ito colourblind-safe palette
OI = {
    "black":  "#000000",
    "orange": "#E69F00",
    "skyblue": "#56B4E9",
    "green":  "#009E73",
    "yellow": "#F0E442",
    "blue":   "#0072B2",
    "vermil": "#D55E00",
    "purple": "#CC79A7",
    "grey":   "#999999",
}

plt.rcParams.update({
    "figure.facecolor": "white",
    "savefig.facecolor": "white",
    "axes.facecolor": "white",
    "font.family": "sans-serif",
    "font.size": 9,
    "axes.labelsize": 9,
    "axes.titlesize": 10,
    "xtick.labelsize": 8,
    "ytick.labelsize": 8,
    "legend.frameon": False,
    "axes.spines.top": False,
    "axes.spines.right": False,
})

# Column mapping: source workbook header -> analysis name used throughout.
COLUMN_MAP = {
    "MFO (g/m)":             "mfo",
    "VO2peak_ABS(L/min)":   "vo2_abs",
    "VO2peak":              "vo2_rel",
    "Age":                  "age",
    "Body.Mass":            "mass",
    "Stature":              "stature",
    "IMC":                  "bmi",
    "BSA":                  "bsa",
    "FFM.DXA":              "ffm_dxa",
    "FM.DXA":               "fm_dxa",
    "FM.Correct.DXa":       "fm_corr",
    "FFAT":                 "ffat",
    "FFM.BIA":              "ffm_bia",
    "FM.BIA":               "fm_bia",
    "Muss.index":           "muscle_index",
    "Neck.CG":              "neck_cg",
    "Arm.CG":               "arm_cg",
    "Chest.CG":             "chest_cg",
    "Waist.CG":             "waist_cg",
    "Thigh.CG":             "thigh_cg",
    "Calf.CG":              "calf_cg",
    "X6CG":                 "sum6cg",
    "Calf.G":               "calf_g",
    "Tórax.G":              "chest_g",
    "Calf.SF":              "sf_calf",
    "Subscapular.SF":       "sf_sub",
    "Supraspinal.SF":       "sf_supra",
    "RHR":                  "rhr",
    "Sex":                  "sex",
    "sport_energy_system":  "energy_system",
    "Cluster":              "cluster_published",
}

# Six ISAK skinfold-corrected girths (Section 2.4 of the main text)
CG6 = ["neck_cg", "arm_cg", "chest_cg", "waist_cg", "thigh_cg", "calf_cg"]

# Variable set entered into the exploratory k-means analysis (Section 3.6).
# All variables are body-size, body-composition or anthropometric descriptors
# measured independently of the outcome; MFO and VO2peak are deliberately
# excluded so that the partition cannot be circular with respect to the
# outcome modelled in Sections 3.3-3.5.
CLUSTER_VARS = (
    ["mass", "stature"]
    + ["ffm_bia", "fm_bia"]
    + CG6
    + ["calf_g", "chest_g"]
    + ["sf_calf", "sf_sub", "sf_supra"]
    + ["fm_corr", "ffat"]
    + ["age", "rhr"]
)

# Published values, hard-coded here only so that the script can verify itself
# at run time (Section 12). They are never used in any computation.
#
# "D" marks deterministic quantities, which reproduce exactly.
# "S" marks stochastic quantities (bootstrap percentile limits, resampling-
#     based stability indices), which depend on the pseudo-random stream of
#     the NumPy build in use and reproduce only to the stated tolerance.
PUBLISHED = [
    # (label, published value, tolerance, kind)
    ("n (analytic sample)",                     43,      0),
    ("n men",                                   21,      0),
    ("n women",                                 22,      0),
    ("Median MFO (g/min)",                      0.730,   0.001),
    ("Median absolute VO2peak (L/min)",         3.19,    0.01),
    ("Median normalised VO2peak (mL/kg/min)",   53.10,   0.01),
    ("Spearman rho, MFO ~ absolute VO2peak",    0.667,   0.001),
    ("Spearman rho, MFO ~ normalised VO2peak", 0.597,   0.001),
    ("Spearman rho, MFO ~ corrected calf girth", 0.496, 0.001),
    ("M1 R2",                                   0.604,   0.001),
    ("M1 adjusted R2",                          0.594,   0.001),
```

```
    ("M1 Q2 (LOOCV)",                          0.563,   0.001),
    ("M1 F statistic",                         62.5,    0.1),
    ("M1 beta, absolute VO2peak",              0.191,   0.001),
    ("M1 AIC",                                 -45.2,   0.1),
    ("M1 Breusch-Pagan p",                     0.900,   0.005),
    ("M1 RESET p",                             0.210,   0.005),
    ("M1 Durbin-Watson",                       2.01,    0.01),
    ("M1 Shapiro-Wilk p (residuals)",          0.009,   0.001),
    ("M1 n Cook's D above 4/n",                2,       0),
    ("Allometric scaling exponent",            0.865,   0.001),
    ("S1 delta R2 (corrected calf girth)",     0.006,   0.001),
    ("S1 beta (corrected calf girth)",         0.0092,  0.0001),
    ("S1 p (corrected calf girth)",            0.424,   0.001),
    ("S1 Q2",                                  0.541,   0.001),
    ("S1 delta AIC",                           1.3,     0.1),
    ("S7 beta (fat-free mass, DXA)",           -0.0045, 0.0001),
    ("S7 p (fat-free mass, DXA)",              0.268,   0.001),
    ("S5 p (corrected calf girth)",            0.244,   0.001),
    ("S5 p (fat-free mass)",                   0.165,   0.001),
    ("S11 R2 (outcome scaled to FFM)",         0.032,   0.001),
    ("S11 Q2 (outcome scaled to FFM)",         -0.106,  0.001),
    ("Normalised specification R2",            0.415,   0.001),
    ("Normalised specification Q2",            0.338,   0.001),
    ("Normalised specification AIC",           -28.4,   0.1),
    ("Normalised + calf girth, beta",          0.0366,  0.0001),
    ("Normalised + calf girth, R2",            0.569,   0.001),
    ("Men: R2",                                0.760,   0.001),
    ("Men: Q2",                                0.702,   0.001),
    ("Men: beta",                              0.2192,  0.0001),
    ("Women: R2",                              0.168,   0.001),
    ("Women: Q2",                              0.024,   0.001),
    ("Women: beta",                            0.1397,  0.0001),
    ("Women: p",                               0.058,   0.001),
    ("Mean silhouette width (k = 2)",          0.286,   0.001),
    ("Mean silhouette width (k = 3)",          0.204,   0.001),
    ("Cluster 1 size",                         27,      0),
    ("Cluster 2 size",                         16,      0),
    ("Women in cluster 2",                     1,       0),
    ("Cramer's V, cluster vs sex",             0.64,    0.01),
    ("Bootstrap Jaccard stability, cluster 1", 0.93,    0.03),
    ("Bootstrap Jaccard stability, cluster 2", 0.89,    0.03),
]

# Quantities in PUBLISHED whose value depends on the pseudo-random stream.
STOCHASTIC = {
    "Bootstrap Jaccard stability, cluster 1",
    "Bootstrap Jaccard stability, cluster 2",
}

LOG_LINES: list[str] = []



def log(msg: str = "") -> None:
    print(msg)
    LOG_LINES.append(str(msg))



# ============================================================================
# 1. HELPER FUNCTIONS
# ============================================================================


def median_iqr(x: np.ndarray, dec: int = 2) -> str:
    """Median (25th-75th percentile), the descriptive format used in Table 1."""
    x = np.asarray(x, float)
    return (f"{np.median(x):.{dec}f} "
            f"({np.percentile(x, 25):.{dec}f}-{np.percentile(x, 75):.{dec}f})")



def probability_of_superiority(a, b) -> float:
    """P(X > Y) + 0.5 P(X = Y); the common-language effect size for the
    Mann-Whitney U test. Values above 0.5 favour group `a`."""
    a, b = np.asarray(a, float), np.asarray(b, float)
    u = stats.mannwhitneyu(a, b, alternative="two-sided").statistic
    return float(u / (len(a) * len(b)))



def bh_fdr(pvals) -> np.ndarray:
    """Benjamini-Hochberg adjusted p values (false discovery rate)."""
    return multipletests(np.asarray(pvals, float), method="fdr_bh")[1]



def fit_ols(data: pd.DataFrame, predictors: list[str], outcome: str = "mfo"):
    """Ordinary least squares with an intercept. Returns (result, X, y)."""
    y = np.asarray(data[outcome], float)
    X = np.asarray(data[predictors], float)
    Xd = sm.add_constant(X, has_constant="add")
    return sm.OLS(y, Xd).fit(), Xd, y



def loocv_q2(result, y) -> float:
    """Leave-one-out cross-validated R^2 (Q^2 = 1 - PRESS/TSS).

    Computed in closed form from the hat-matrix diagonal, which is
    algebraically identical to refitting the model n times."""
    h = result.get_influence().hat_matrix_diag
    press = float(np.sum((result.resid / (1.0 - h)) ** 2))
    tss = float(np.sum((y - y.mean()) ** 2))
    return 1.0 - press / tss



def max_vif(Xd: np.ndarray) -> float:
    """Largest variance inflation factor among the predictors (excluding the
    intercept). Returns 1.0 for a single-predictor model."""
    if Xd.shape[1] <= 2:
```

```python
        return 1.0
    return float(max(variance_inflation_factor(Xd, i)
                     for i in range(1, Xd.shape[1])))


def bootstrap_coef_ci(data, predictors, outcome="mfo", index=1,
                      n_boot=N_BOOT, seed=SEED):
    """Percentile bootstrap confidence interval for a regression coefficient
    (case resampling). Used because the residuals depart from normality."""
    rng = np.random.default_rng(seed)
    y = np.asarray(data[outcome], float)
    X = np.asarray(data[predictors], float)
    n = len(y)
    draws = np.empty(n_boot)
    for b in range(n_boot):
        idx = rng.integers(0, n, n)
        Xd = sm.add_constant(X[idx], has_constant="add")
        draws[b] = sm.OLS(y[idx], Xd).fit().params[index]
    return float(np.percentile(draws, 2.5)), float(np.percentile(draws, 97.5))


def bootstrap_spearman_ci(x, y, n_boot=N_BOOT, seed=SEED):
    """Percentile bootstrap confidence interval for Spearman's rho."""
    rng = np.random.default_rng(seed)
    x, y = np.asarray(x, float), np.asarray(y, float)
    n = len(x)
    draws = np.empty(n_boot)
    for b in range(n_boot):
        idx = rng.integers(0, n, n)
        draws[b] = stats.spearmanr(x[idx], y[idx]).statistic
    return float(np.percentile(draws, 2.5)), float(np.percentile(draws, 97.5))


def fisher_z_ci(rho: float, n: int, alpha: float = 0.05):
    """Confidence interval for a correlation via Fisher's z transformation.
    Used for the attainable-precision analysis (Table 6, Figure A2)."""
    z = np.arctanh(rho)
    se = 1.0 / np.sqrt(n - 3)
    crit = stats.norm.ppf(1 - alpha / 2)
    return float(np.tanh(z - crit * se)), float(np.tanh(z + crit * se))


def fmt_p(p: float) -> str:
    return "<0.001" if p < 0.001 else f"{p:.3f}"


# ============================================================================
# 2. DATA LOADING AND DERIVED VARIABLES
# ============================================================================


def load_data(path: Path) -> pd.DataFrame:
    raw = pd.read_excel(path)
    missing = [c for c in COLUMN_MAP if c not in raw.columns]
    if missing:
        raise KeyError(f"Expected column(s) not found in {path.name}: {missing}")


    d = raw.rename(columns=COLUMN_MAP)[list(COLUMN_MAP.values())].copy()


    # --- Derived variables ---------------------------------------------------
    # MFO expressed per kilogram of DXA-derived fat-free mass, in mg/min/kg.
    d["mfo_per_ffm"] = d["mfo"] / d["ffm_dxa"] * 1000.0
    # Natural-log transforms for the allometric specification (Model A1).
    d["ln_mfo"] = np.log(d["mfo"])
    d["ln_vo2_abs"] = np.log(d["vo2_abs"])
    # Binary contrast used in sensitivity model S13: sports whose predominant
    # energy system is oxidative (code 1) versus all other sports (codes 2-4).
    d["oxidative_sport"] = (d["energy_system"] == 1).astype(int)
    # Sex is coded 1 = men, 2 = women in the source workbook.
    d["male"] = (d["sex"] == 1).astype(int)


    log("=" * 78)
    log("DATA")
    log("=" * 78)
    log(f"File                : {path.name}")
    log(f"Rows x columns      : {d.shape[0]} x {raw.shape[1]}")
    log(f"Missing values      : {int(d.isna().sum().sum())}")
    log(f"Men / women         : {int((d.sex == 1).sum())} / {int((d.sex == 2).sum())}")
    es = d["energy_system"].value_counts().sort_index()
    log("Energy system (n)   : oxidative %d, glycolytic %d, phosphagen %d, "
        "intermittent %d" % (es.get(1, 0), es.get(2, 0), es.get(3, 0), es.get(4, 0)))
    log("")
    return d


# ============================================================================
# 2b. DATA-INTEGRITY CHECKS
# ============================================================================
#
# The columns of the workbook are not all mutually independent: several are
# arithmetic functions of others. These checks recompute each derived column
# from its stated inputs and report any disagreement. They do not alter any
# analysis; they exist so that a reader can confirm the internal consistency
# of the deposited dataset, and so that any discrepancy is visible rather than
# silent.
# ============================================================================


def data_integrity_checks(d: pd.DataFrame, outdir: Path) -> pd.DataFrame:
    rows = []


    def check(name, expected, observed, tol, note=""):
        diff = np.abs(np.asarray(expected, float) - np.asarray(observed, float))
        n_bad = int((diff > tol).sum())
        rows.append({"Check": name,
                     "Max absolute difference": round(float(diff.max()), 4),
```

```
                    "Rows exceeding tolerance": n_bad,
                    "Status": "CONSISTENT" if n_bad == 0 else "DISCREPANT",
                    "Note": note})
        return diff


    # Body mass index = body mass / stature^2
    check("BMI = body mass / stature^2",
          d["mass"] / (d["stature"] / 100) ** 2, d["bmi"], 1e-6)


    # Normalised VO2peak = absolute VO2peak / body mass
    check("Normalised VO2peak = absolute VO2peak / body mass x 1000",
          d["vo2_abs"] / d["mass"] * 1000, d["vo2_rel"], 1e-6)


    # Muscle index = DXA fat-free mass / stature^2 (fat-free mass index)
    check("Muscle index = DXA fat-free mass / stature^2",
          d["ffm_dxa"] / (d["stature"] / 100) ** 2, d["muscle_index"], 1e-6)


    # Fat-free adipose tissue is a fixed fraction of DXA fat mass
    ratio = float(np.median(d["ffat"] / d["fm_dxa"]))
    check(f"Fat-free adipose tissue = {ratio:.4f} x DXA fat mass",
          ratio * d["fm_dxa"], d["ffat"], 1e-3)


    # Corrected fat mass = DXA fat mass + fat-free adipose tissue
    check("Corrected fat mass = DXA fat mass + fat-free adipose tissue",
          d["fm_dxa"] + d["ffat"], d["fm_corr"], 1e-6)


    # Corrected calf girth = calf girth - pi x (medial calf skinfold / 10)
    check("Corrected calf girth = calf girth - pi x (calf skinfold / 10)",
          d["calf_g"] - np.pi * d["sf_calf"] / 10.0, d["calf_cg"], 1e-6)


    # Corrected chest girth = chest girth - pi x (subscapular skinfold / 10)
    check("Corrected chest girth = chest girth - pi x (subscapular SF / 10)",
          d["chest_g"] - np.pi * d["sf_sub"] / 10.0, d["chest_cg"], 0.05,
          "Recheck the subscapular skinfold for the discrepant record(s)")


    # Sum of six corrected girths
    check("Sum of six corrected girths = sum of the six columns",
          d[CG6].sum(axis=1), d["sum6cg"], 0.05,
          "Recheck the arithmetic for the discrepant record(s)")


    # Body surface area: the supplied column should be a function of body mass
    # and stature. Du Bois is used as the reference equation.
    bsa_dubois = 0.007184 * d["mass"] ** 0.425 * d["stature"] ** 0.725
    check("Body surface area = Du Bois equation (record by record)",
          bsa_dubois, d["bsa"], 0.01,
          "See the note below")


    integ = pd.DataFrame(rows)
    integ.to_csv(outdir / "data_integrity_checks.csv", index=False)


    log("=" * 78)
    log("DATA-INTEGRITY CHECKS")
    log("=" * 78)
    log(integ.to_string(index=False))
    log("")


    # --- Detailed note on the body-surface-area column -----------------------
    sorted_gap = float(np.abs(np.sort(np.asarray(d["bsa"], float))
                              - np.sort(np.asarray(bsa_dubois, float))).max())
    r_supplied = stats.spearmanr(d["mfo"], d["bsa"])
    r_dubois = stats.spearmanr(d["mfo"], bsa_dubois)
    log("Note on the body-surface-area column")
    log("-" * 78)
    log("The supplied BSA column does not agree with the Du Bois equation")
    log("record by record, yet the two sets of values agree almost exactly once")
    log("each is sorted independently (largest gap between the sorted sets: "
        f"{sorted_gap:.4f} m2).")
    log("The column therefore contains the correct Du Bois values, but they are")
    log("not aligned to the correct participants. Consequences:")
    log(f"  Spearman rho with MFO, column as supplied : {r_supplied.statistic:+.3f} "
        f"(p = {r_supplied.pvalue:.3f})")
    log(f"  Spearman rho with MFO, recomputed Du Bois : {r_dubois.statistic:+.3f} "
        f"(p = {r_dubois.pvalue:.3f})")
    log("Body surface area is not a predictor in any model reported in the")
    log("article, so no model result is affected. Only the corresponding row of")
    log("Table 2 is affected. Table 2 below is produced from the column as")
    log("supplied, so that this script reproduces the published article; the")
    log("recomputed value above is the one that should replace it.")
    log("")
    return integ



# =============================================================================
# 3. TABLE 1 - PARTICIPANT CHARACTERISTICS
# =============================================================================


TABLE1_ROWS = [
    ("age",           "Age (y)", 1),
    ("mass",          "Body mass (kg)", 2),
    ("stature",       "Stretch stature (cm)", 2),
    ("bmi",           "Body mass index (kg/m2)", 2),
    ("mfo",           "MFO (g/min)", 3),
    ("mfo_per_ffm",   "MFO per kg FFM (mg/min/kg)", 2),
    ("vo2_abs",       "Absolute VO2peak (L/min)", 2),
    ("vo2_rel",       "Normalised VO2peak (mL/kg/min)", 2),
    ("rhr",           "Resting heart rate (beats/min)", 1),
    ("ffm_dxa",       "Fat-free mass, DXA (kg)", 2),
    ("fm_dxa",        "Fat mass, DXA (kg)", 2),
    ("ffm_bia",       "Fat-free mass, BIA (kg)", 2),
    ("fm_bia",        "Fat mass, BIA (kg)", 2),
    ("calf_g",        "Calf girth, uncorrected (cm)", 2),
    ("calf_cg",       "Corrected calf girth (cm)", 2),
    ("thigh_cg",      "Corrected thigh girth (cm)", 2),
    ("arm_cg",        "Corrected arm girth (cm)", 2),
    ("neck_cg",       "Corrected neck girth (cm)", 2),
    ("chest_cg",      "Corrected chest girth (cm)", 2),
```

```
    ("waist_cg",     "Corrected waist girth (cm)", 2),
    ("sum6cg",       "Sum of six corrected girths (cm)", 2),
]



def table1(d: pd.DataFrame, outdir: Path) -> pd.DataFrame:
    men, women = d[d.sex == 1], d[d.sex == 2]
    rows, pvals = [], []
    for var, label, dec in TABLE1_ROWS:
        p = stats.mannwhitneyu(men[var], women[var],
                               alternative="two-sided").pvalue
        rows.append({
            "Variable": label,
            f"Total (n = {len(d)})": median_iqr(d[var], dec),
            f"Men (n = {len(men)})": median_iqr(men[var], dec),
            f"Women (n = {len(women)})": median_iqr(women[var], dec),
            "PS": f"{probability_of_superiority(men[var], women[var]):.2f}",
            "p": fmt_p(p),
        })
        pvals.append(p)
    q = bh_fdr(pvals)
    for r, qi in zip(rows, q):
        r["q"] = fmt_p(qi)
    t1 = pd.DataFrame(rows)
    t1.to_csv(outdir / "table1_participant_characteristics.csv", index=False)


    log("=" * 78)
    log("TABLE 1 - Participant characteristics (median [IQR]; Mann-Whitney U;")
    log("           Benjamini-Hochberg FDR within this family)")
    log("=" * 78)
    log(t1.to_string(index=False))
    log("")
    return t1



# ============================================================================
# 4. TABLE 2 AND FIGURE 2 - UNADJUSTED CORRELATIONS WITH MFO
# ============================================================================

CORR_VARS = [
    ("vo2_abs",      "Absolute VO2peak (L/min)"),
    ("vo2_rel",      "Normalised VO2peak (mL/kg/min)"),
    ("calf_cg",      "Corrected calf girth"),
    ("calf_g",       "Calf girth (uncorrected)"),
    ("ffm_dxa",      "Fat-free mass (DXA)"),
    ("muscle_index", "Muscle index"),
    ("ffm_bia",      "Fat-free mass (BIA)"),
    ("neck_cg",      "Corrected neck girth"),
    ("thigh_cg",     "Corrected thigh girth"),
    ("mass",         "Body mass"),
    ("arm_cg",       "Corrected arm girth"),
    ("sum6cg",       "Sum of six corrected girths"),
    ("stature",      "Stretch stature"),
    ("chest_g",      "Chest girth (uncorrected)"),
    ("chest_cg",     "Corrected chest girth"),
    ("fm_dxa",       "Fat mass (DXA)"),
    ("fm_corr",      "Corrected fat mass"),
    ("ffat",         "Fat-free adipose tissue"),
    ("age",          "Age"),
    ("bmi",          "Body mass index"),
    ("waist_cg",     "Corrected waist girth"),
    ("sf_sub",       "Subscapular skinfold"),
    ("fm_bia",       "Fat mass (BIA)"),
    ("sf_calf",      "Medial calf skinfold"),
    ("sf_supra",     "Supraspinale skinfold"),
    ("rhr",          "Resting heart rate"),
    ("bsa",          "Body surface area (column as supplied)"),
]



def table2_figure2(d: pd.DataFrame, outdir: Path) -> pd.DataFrame:
    rows, pvals = [], []
    for i, (var, label) in enumerate(CORR_VARS):
        rho, p = stats.spearmanr(d["mfo"], d[var])
        # A distinct but deterministic seed per variable keeps every interval
        # independent of the order in which variables are processed.
        lo, hi = bootstrap_spearman_ci(d["mfo"], d[var], seed=SEED + i)
        rows.append({"Variable": label, "rho": round(float(rho), 3),
                     "CI_low": round(lo, 2), "CI_high": round(hi, 2),
                     "p": p})
        pvals.append(p)
    t2 = pd.DataFrame(rows)
    t2["q"] = bh_fdr(pvals)
    t2 = t2.sort_values("rho", key=lambda s: s.abs(), ascending=False)
    t2["95% CI"] = [f"{lo:.2f} to {hi:.2f}"
                    for lo, hi in zip(t2.CI_low, t2.CI_high)]
    out = t2[["Variable", "rho", "95% CI", "p", "q"]].copy()
    out["p"] = out["p"].map(fmt_p)
    out["q"] = out["q"].map(fmt_p)
    out.to_csv(outdir / "table2_unadjusted_correlations.csv", index=False)


    log("=" * 78)
    log("TABLE 2 - Unadjusted Spearman correlations with absolute MFO (n = 43)")
    log("           95% CI from 5000 bootstrap resamples; BH-FDR across the table")
    log("=" * 78)
    log(out.to_string(index=False))
    log("")

    # --- Figure 2 -----------------------------------------------------------
    plot = t2.iloc[::-1].reset_index(drop=True)
    sig = plot["q"] < 0.05
    fig, ax = plt.subplots(figsize=(6.0, 7.2))
    ypos = np.arange(len(plot))
    colors = [OI["blue"] if s else OI["grey"] for s in sig]
    for i in range(len(plot)):
        ax.plot([plot["CI_low"][i], plot["CI_high"][i]], [ypos[i], ypos[i]],
                color=colors[i], lw=1.1, solid_capstyle="butt", zorder=2)
    ax.scatter(plot["rho"], ypos, c=colors, s=22, zorder=3)
    ax.axvline(0, color=OI["black"], lw=0.8, ls="--")
```

```
    ax.set_yticks(ypos)
    ax.set_yticklabels(plot["Variable"])
    ax.set_xlabel("Spearman's rho with absolute MFO (g/min)")
    ax.set_xlim(-0.80, 1.00)
    ax.set_ylim(-1, len(plot))
    ax.text(0.98, 0.10, "Significant after\nBH-FDR control", color=OI["blue"],
            fontsize=8, ha="right", va="bottom", transform=ax.transAxes)
    ax.text(0.98, 0.02, "Not significant", color=OI["grey"], fontsize=8,
            ha="right", va="bottom", transform=ax.transAxes)
    fig.tight_layout()
    fig.savefig(outdir / "figure2_correlations.png", dpi=DPI)
    plt.close(fig)
    return t2


# ============================================================================
# 5. PRIMARY MODEL, DIAGNOSTICS AND FIGURE 3
# ============================================================================

def primary_model(d: pd.DataFrame, outdir: Path) -> dict:
    res, Xd, y = fit_ols(d, ["vo2_abs"])
    q2 = loocv_q2(res, y)
    ci = res.conf_int()[1]
    boot_lo, boot_hi = bootstrap_coef_ci(d, ["vo2_abs"], index=1)

    bp_p = het_breuschpagan(res.resid, Xd)[1]
    reset_p = linear_reset(res, power=2, use_f=True).pvalue
    dw = durbin_watson(res.resid)
    sw_p = stats.shapiro(res.resid).pvalue
    cooks = res.get_influence().cooks_distance[0]
    n_infl = int((cooks > 4 / len(y)).sum())

    # Robust regression (Huber weighting) as a further check.
    rlm = sm.RLM(y, Xd, M=sm.robust.norms.HuberT()).fit()

    # Leave-one-out influence on the coefficient of interest.
    loo_betas = []
    for i in range(len(y)):
        keep = np.ones(len(y), bool); keep[i] = False
        loo_betas.append(sm.OLS(y[keep], Xd[keep]).fit().params[1])
    loo_betas = np.asarray(loo_betas)

    # Allometric model A1: ln(MFO) ~ ln(absolute VO2peak)
    allo, _, ly = fit_ols(d, ["ln_vo2_abs"], outcome="ln_mfo")
    allo_ci = allo.conf_int()[1]

    log("=" * 78)
    log("PRIMARY MODEL M1 - absolute MFO (g/min) ~ absolute VO2peak (L/min)")
    log("=" * 78)
    log(f"n                    : {len(y)}")
    log(f"R2                   : {res.rsquared:.3f}")
    log(f"Adjusted R2          : {res.rsquared_adj:.3f}")
    log(f"Q2 (LOOCV)           : {q2:.3f}")
    log(f"F(1, {int(res.df_resid)})              : {res.fvalue:.1f}, p = {fmt_p(res.f_pvalue)}")
    log(f"Intercept            : {res.params[0]:.4f}")
    log(f"Beta (VO2peak)       : {res.params[1]:.4f} "
        f"(95% CI {ci[0]:.3f} to {ci[1]:.3f})")
    log(f"Bootstrap 95% CI     : {boot_lo:.3f} to {boot_hi:.3f} "
        f"({N_BOOT} resamples)")
    log(f"AIC                  : {res.aic:.1f}")
    log("")
    log("Diagnostics")
    log(f"  Breusch-Pagan p    : {bp_p:.3f}")
    log(f"  Ramsey RESET p     : {reset_p:.3f}")
    log(f"  Durbin-Watson      : {dw:.2f}")
    log(f"  Shapiro-Wilk p     : {sw_p:.3f}  (residuals)")
    log(f"  Cook's D > 4/n     : {n_infl} observation(s)")
    log(f"  Robust (Huber) beta : {rlm.params[1]:.4f}")
    log(f"  Leave-one-out beta  : {loo_betas.min():.4f} to {loo_betas.max():.4f}")
    log("")
    log("Allometric model A1 - ln(MFO) ~ ln(absolute VO2peak)")
    log(f"  Scaling exponent    : {allo.params[1]:.3f} "
        f"(95% CI {allo_ci[0]:.3f} to {allo_ci[1]:.3f})")
    log(f"  R2 / Q2             : {allo.rsquared:.3f} / {loocv_q2(allo, ly):.3f}")
    log("")

    with open(outdir / "primary_model_diagnostics.txt", "w") as fh:
        fh.write(str(res.summary()))
        fh.write("\n\nRobust regression (Huber M-estimator)\n")
        fh.write(str(rlm.summary()))

    # --- Figure 3 ----------------------------------------------------------
    fig, axes = plt.subplots(1, 3, figsize=(10.5, 3.5))

    def scatter_fit(ax, xvar, xlabel, title):
        x = np.asarray(d[xvar], float)
        r, _, _ = fit_ols(d, [xvar])
        xs = np.linspace(x.min(), x.max(), 200)
        pred = r.get_prediction(sm.add_constant(xs.reshape(-1, 1))).summary_frame()
        ax.fill_between(xs, pred["mean_ci_lower"], pred["mean_ci_upper"],
                        color=OI["skyblue"], alpha=0.30, lw=0)
        ax.plot(xs, pred["mean"], color=OI["blue"], lw=1.6)
        ax.scatter(x[d.male == 1], d["mfo"][d.male == 1], s=20,
                   color=OI["blue"], label="Men", zorder=3)
        ax.scatter(x[d.male == 0], d["mfo"][d.male == 0], s=20,
                   facecolors="none", edgecolors=OI["orange"], linewidths=1.0,
                   label="Women", zorder=3)
        ax.set_xlabel(xlabel)
        ax.set_ylabel("Absolute MFO (g/min)")
        ax.set_title(title, loc="left", fontweight="bold")
        ax.text(0.03, 0.94, f"$R^2$ = {r.rsquared:.3f}\n$Q^2$ = {loocv_q2(r, np.asarray(d['mfo'], float)):.3f}",
                transform=ax.transAxes, va="top", fontsize=8)

    scatter_fit(axes[0], "vo2_abs", "Absolute VO$_2$peak (L/min)",
                "(A) Dimensionally matched")
    axes[0].legend(loc="lower right", fontsize=8)
    scatter_fit(axes[1], "vo2_rel", "Normalised VO$_2$peak (mL/kg/min)",
                "(B) Size-normalised predictor")
```

```
    # (C) Added-variable plot: corrected calf girth given absolute VO2peak
    ry = sm.OLS(np.asarray(d["mfo"], float),
                sm.add_constant(np.asarray(d[["vo2_abs"]], float))).fit().resid
    rx = sm.OLS(np.asarray(d["calf_cg"], float),
                sm.add_constant(np.asarray(d[["vo2_abs"]], float))).fit().resid
    av = sm.OLS(ry, sm.add_constant(rx)).fit()
    xs = np.linspace(rx.min(), rx.max(), 200)
    pred = av.get_prediction(sm.add_constant(xs.reshape(-1, 1))).summary_frame()
    axes[2].fill_between(xs, pred["mean_ci_lower"], pred["mean_ci_upper"],
                         color=OI["vermil"], alpha=0.22, lw=0)
    axes[2].plot(xs, pred["mean"], color=OI["vermil"], lw=1.6)
    axes[2].scatter(rx, ry, s=20, color=OI["black"], zorder=3)
    axes[2].axhline(0, color=OI["grey"], lw=0.8, ls=":")
    axes[2].set_xlabel("Corrected calf girth | absolute VO$_2$peak (cm)")
    axes[2].set_ylabel("Absolute MFO | absolute VO$_2$peak (g/min)")
    axes[2].set_title("(C) Added-variable plot", loc="left", fontweight="bold")
    s1, _, _ = fit_ols(d, ["vo2_abs", "calf_cg"])
    axes[2].text(0.03, 0.94,
                 f"$\\beta$ = {s1.params[2]:+.4f}\np = {s1.pvalues[2]:.3f}",
                 transform=axes[2].transAxes, va="top", fontsize=8)

    fig.tight_layout()
    fig.savefig(outdir / "figure3_specification.png", dpi=DPI)
    plt.close(fig)

    return {"res": res, "q2": q2, "aic": float(res.aic), "y": y,
            "bp_p": float(bp_p), "reset_p": float(reset_p), "dw": float(dw),
            "sw_p": float(sw_p), "n_infl": n_infl,
            "allo_exp": float(allo.params[1])}


# ============================================================================
# 6. TABLE 3 AND FIGURE 4 - PRE-SPECIFIED SENSITIVITY MODELS
# ============================================================================

MODELS = [
    ("M1",  "Absolute VO2peak (primary)",                                ["vo2_abs"], "mfo"),
    ("S1",  "M1 + corrected calf girth",                                 ["vo2_abs", "calf_cg"], "mfo"),
    ("S9",  "M1 + uncorrected calf girth",                               ["vo2_abs", "calf_g"], "mfo"),
    ("S7",  "M1 + fat-free mass",                                        ["vo2_abs", "ffm_dxa"], "mfo"),
    ("S5",  "M1 + corrected calf girth + fat-free mass",                 ["vo2_abs", "calf_cg", "ffm_dxa"], "mfo"),
    ("S4",  "M1 + corrected calf girth + body mass",                     ["vo2_abs", "calf_cg", "mass"], "mfo"),
    ("S3",  "M1 + corrected calf girth + stature",                       ["vo2_abs", "calf_cg", "stature"], "mfo"),
    ("S2",  "M1 + corrected calf girth + sex",                           ["vo2_abs", "calf_cg", "male"], "mfo"),
    ("S6",  "M1 + corrected calf girth + sex + stature + fat-free mass", ["vo2_abs", "calf_cg", "male", "stature",
"ffm_dxa"], "mfo"),
    ("S13", "M1 + sport energy system",                                  ["vo2_abs", "oxidative_sport"], "mfo"),
    ("S15", "M1 + age + resting heart rate",                             ["vo2_abs", "age", "rhr"], "mfo"),
    ("A1",  "Allometric log-log model",                                  ["ln_vo2_abs"], "ln_mfo"),
    ("S11", "Outcome scaled to fat-free mass + corrected calf girth",    ["vo2_abs", "calf_cg"], "mfo_per_ffm"),
]

# Additional single-covariate sensitivity models reported in the text only.
EXTRA_COVARIATES = [
    ("fat mass (DXA)",         "fm_dxa"),
    ("sex",                    "male"),
    ("stature",                "stature"),
    ("body mass",              "mass"),
    ("age",                    "age"),
    ("resting heart rate",     "rhr"),
]


def table3_figure4(d: pd.DataFrame, base_aic: float, outdir: Path) -> pd.DataFrame:
    rows = []
    for tag, label, preds, outcome in MODELS:
        res, Xd, y = fit_ols(d, preds, outcome=outcome)
        rows.append({
            "Model": f"{tag}. {label}",
            "k": len(preds),
            "R2": round(float(res.rsquared), 3),
            "Adj. R2": round(float(res.rsquared_adj), 3),
            "Q2": round(loocv_q2(res, y), 3),
            "AIC": round(float(res.aic), 1),
            "dAIC": round(float(res.aic) - base_aic, 1),
            "Max VIF": round(max_vif(Xd), 2),
        })
    t3 = pd.DataFrame(rows)
    t3.to_csv(outdir / "table3_models.csv", index=False)

    log("=" * 78)
    log("TABLE 3 - Primary and sensitivity models for absolute MFO (n = 43)")
    log("=" * 78)
    log(t3.to_string(index=False))
    log("")

    log("Single-covariate sensitivity models added to M1 (reported in text)")
    log("-" * 78)
    r7, _, _ = fit_ols(d, ["vo2_abs", "ffm_dxa"])
    log(f"  fat-free mass (DXA)  beta = {r7.params[2]:+.4f}, p = {r7.pvalues[2]:.3f}")
    for label, var in EXTRA_COVARIATES:
        r, _, _ = fit_ols(d, ["vo2_abs", var])
        log(f"  {label:20s} beta = {r.params[2]:+.4f}, p = {r.pvalues[2]:.3f}")
    r13, _, _ = fit_ols(d, ["vo2_abs", "oxidative_sport"])
    log(f"  {'sport energy system':20s} beta = {r13.params[2]:+.4f}, "
        f"p = {r13.pvalues[2]:.3f}")
    r5, _, _ = fit_ols(d, ["vo2_abs", "calf_cg", "ffm_dxa"])
    log(f"  S5 (both morphological terms): p(calf girth) = {r5.pvalues[2]:.3f}, "
        f"p(FFM) = {r5.pvalues[3]:.3f}")
    log("")

    # --- Figure 4 ----------------------------------------------------------
    plot = t3[~t3.Model.str.startswith(("A1", "S11"))].copy()
    labels = [m.split(".")[0] for m in plot.Model]
    x = np.arange(len(plot))
    fig, axes = plt.subplots(1, 2, figsize=(10.0, 3.8))
    w = 0.38
```

```
    axes[0].bar(x - w / 2, plot["R2"], w, color=OI["blue"], label="$R^2$ (apparent)")
    axes[0].bar(x + w / 2, plot["Q2"], w, color=OI["orange"], label="$Q^2$ (LOOCV)")
    axes[0].set_xticks(x); axes[0].set_xticklabels(labels, rotation=0)
    axes[0].set_ylabel("Explained variance")
    axes[0].set_ylim(0, 0.80)
    axes[0].set_title("(A) Apparent and cross-validated fit", loc="left",
                      fontweight="bold")
    axes[0].legend(loc="upper right", fontsize=8)

    cols = [OI["vermil"] if v > 0 else OI["green"] for v in plot["dAIC"]]
    axes[1].bar(x, plot["dAIC"], 0.6, color=cols)
    axes[1].axhline(0, color=OI["black"], lw=0.8)
    axes[1].set_xticks(x); axes[1].set_xticklabels(labels, rotation=0)
    axes[1].set_ylabel("$\\Delta$AIC vs primary model")
    axes[1].set_title("(B) Information criterion (positive = worse)", loc="left",
                      fontweight="bold")
    fig.tight_layout()
    fig.savefig(outdir / "figure4_model_comparison.png", dpi=DPI)
    plt.close(fig)
    return t3


# ============================================================================
# 7. TABLE 4 - INCREMENTAL CONTRIBUTION OF REGIONAL ANTHROPOMETRY
# ============================================================================

INCREMENTAL_VARS = [
    ("calf_cg",  "Corrected calf girth"),
    ("thigh_cg", "Corrected thigh girth"),
    ("arm_cg",   "Corrected arm girth"),
    ("neck_cg",  "Corrected neck girth"),
    ("chest_cg", "Corrected chest girth"),
    ("waist_cg", "Corrected waist girth"),
    ("sum6cg",   "Sum of six corrected girths"),
    ("calf_g",   "Calf girth (uncorrected)"),
]


def table4(d: pd.DataFrame, base_r2: float, outdir: Path) -> pd.DataFrame:
    rows, pvals = [], []
    for i, (var, label) in enumerate(INCREMENTAL_VARS):
        res, _, y = fit_ols(d, ["vo2_abs", var])
        ci = res.conf_int()[2]
        lo, hi = bootstrap_coef_ci(d, ["vo2_abs", var], index=2, seed=SEED + i)
        rows.append({
            "Variable added": label,
            "beta (g/min per unit)": round(float(res.params[2]), 4),
            "95% CI": f"{ci[0]:.4f} to {ci[1]:.4f}",
            "Bootstrap 95% CI": f"{lo:.4f} to {hi:.4f}",
            "dR2": round(float(res.rsquared) - base_r2, 3),
            "Q2": round(loocv_q2(res, y), 3),
            "p": float(res.pvalues[2]),
        })
        pvals.append(res.pvalues[2])
    t4 = pd.DataFrame(rows)
    t4["q"] = np.round(bh_fdr(pvals), 3)
    t4["p"] = t4["p"].round(3)
    t4.to_csv(outdir / "table4_incremental_anthropometry.csv", index=False)

    log("=" * 78)
    log("TABLE 4 - Incremental contribution of regional anthropometric variables")
    log("          added individually to the primary model")
    log("          (BH-FDR across the eight variables in this family)")
    log("=" * 78)
    log(t4.to_string(index=False))
    log("")
    return t4


# ============================================================================
# 8. SIZE-NORMALISED SPECIFICATION (SECTION 3.5)
# ============================================================================

def normalised_specification(d: pd.DataFrame, outdir: Path) -> dict:
    r1, _, y = fit_ols(d, ["vo2_rel"])
    r2, _, _ = fit_ols(d, ["vo2_rel", "calf_cg"])
    ci = r2.conf_int()[2]
    lo, hi = bootstrap_coef_ci(d, ["vo2_rel", "calf_cg"], index=2)

    log("=" * 78)
    log("SECTION 3.5 - Consequences of the size-normalised specification")
    log("=" * 78)
    log(f"MFO ~ normalised VO2peak            : R2 = {r1.rsquared:.3f}, "
        f"Q2 = {loocv_q2(r1, y):.3f}, AIC = {r1.aic:.1f}")
    log(f"MFO ~ normalised VO2peak + calf CG  : R2 = {r2.rsquared:.3f}, "
        f"Q2 = {loocv_q2(r2, y):.3f}, AIC = {r2.aic:.1f}")
    log(f"  beta (corrected calf girth)       : {r2.params[2]:+.4f} "
        f"(95% CI {ci[0]:.4f} to {ci[1]:.4f}; bootstrap {lo:.4f} to {hi:.4f})")
    log(f"  p (corrected calf girth)          : {fmt_p(r2.pvalues[2])}")
    log("")
    log("  Interpretation: the apparently independent contribution of a limb")
    log("  girth is present only when body size has been divided out of the")
    log("  aerobic-capacity term, and disappears when outcome and predictor")
    log("  are expressed on the same dimensional scale.")
    log("")
    return {"r2_norm": float(r1.rsquared), "q2_norm": loocv_q2(r1, y),
            "aic_norm": float(r1.aic), "beta_cg": float(r2.params[2]),
            "r2_two": float(r2.rsquared)}


# ============================================================================
# 9. TABLE 5 - EXPLORATORY SEX-STRATIFIED MODELS
# ============================================================================

def table5(d: pd.DataFrame, outdir: Path) -> pd.DataFrame:
    rows = []
```

```
    for code, label in [(1, "Men"), (2, "Women")]:
        sub = d[d.sex == code]
        res, _, y = fit_ols(sub, ["vo2_abs"])
        ci = res.conf_int()[1]
        res_cg, _, _ = fit_ols(sub, ["vo2_abs", "calf_cg"])
        rows.append({
            "Stratum": f"{label} (n = {len(sub)})",
            "beta (VO2peak)": round(float(res.params[1]), 4),
            "95% CI": f"{ci[0]:.4f} to {ci[1]:.4f}",
            "R2": round(float(res.rsquared), 3),
            "Q2": round(loocv_q2(res, y), 3),
            "p": fmt_p(res.pvalues[1]),
            "beta (calf girth)": round(float(res_cg.params[2]), 4),
            "p (calf girth)": round(float(res_cg.pvalues[2]), 3),
        })
    t5 = pd.DataFrame(rows)
    t5.to_csv(outdir / "table5_sex_stratified.csv", index=False)

    log("=" * 78)
    log("TABLE 5 - Sex-stratified estimates for the primary model (exploratory)")
    log("          Strata are underpowered; reported as hypothesis-generating")
    log("=" * 78)
    log(t5.to_string(index=False))
    log("")
    return t5


# ============================================================================
# 10. TABLE 6 AND FIGURE A2 - ATTAINABLE PRECISION
# ============================================================================

PRECISION_N = [43, 27, 22, 21, 16]
PRECISION_RHO = [0.30, 0.50, 0.70]


def table6_figureA2(outdir: Path) -> pd.DataFrame:
    rows = []
    for n in PRECISION_N:
        row = {"Sample size": n}
        for rho in PRECISION_RHO:
            lo, hi = fisher_z_ci(rho, n)
            row[f"rho = {rho:.2f}"] = f"{lo:.2f} to {hi:.2f} (width {hi - lo:.2f})"
        rows.append(row)
    t6 = pd.DataFrame(rows)
    t6.to_csv(outdir / "table6_attainable_precision.csv", index=False)

    log("=" * 78)
    log("TABLE 6 - Attainable precision: width of the 95% CI for a correlation")
    log("=" * 78)
    log(t6.to_string(index=False))
    log("")

    ns = np.arange(10, 201)
    fig, ax = plt.subplots(figsize=(5.6, 3.8))
    for rho, col in zip(PRECISION_RHO, [OI["blue"], OI["orange"], OI["green"]]):
        widths = [np.subtract(*fisher_z_ci(rho, n)[::-1]) for n in ns]
        ax.plot(ns, widths, color=col, lw=1.6)
        ax.text(ns[-1], widths[-1], f"  $\\rho$ = {rho:.2f}", color=col,
                va="center", fontsize=8)
    # The relevant sample sizes are too close together to label individually,
    # so they are marked with dotted lines and named in a single annotation.
    for n in PRECISION_N:
        ax.axvline(n, color=OI["grey"], ls=":", lw=0.8)
    ax.text(0.97, 0.95,
            "Dotted lines: n = 16, 21, 22, 27, 43\n"
            "(sample sizes relevant to this study)",
            transform=ax.transAxes, ha="right", va="top", fontsize=7,
            color=OI["grey"])
    ax.set_xlabel("Sample size (n)")
    ax.set_ylabel("Width of the 95% confidence interval")
    ax.set_xlim(5, 215)
    ax.set_ylim(0, 1.05)
    fig.tight_layout()
    fig.savefig(outdir / "figureA2_attainable_precision.png", dpi=DPI)
    plt.close(fig)
    return t6


# ============================================================================
# 11. EXPLORATORY UNSUPERVISED ANALYSIS (TABLES A1-A2, FIGURE A1)
# ============================================================================

def jaccard_stability(X: np.ndarray, labels: np.ndarray,
                      n_boot: int = N_BOOT_STABILITY, seed: int = SEED):
    """Cluster-wise Jaccard stability under nonparametric bootstrap
    resampling (Hennig, 2007), following the logic of the `clusterboot`
    procedure: n observations are resampled with replacement, k-means is
    refitted on the resample, every original observation is then assigned to
    the nearest refitted centroid, and each original cluster is matched to the
    recovered cluster with which it shares the largest Jaccard coefficient.

    This is a stochastic quantity: repeated runs vary by roughly +/-0.02."""
    rng = np.random.default_rng(seed)
    n = len(X)
    k = int(labels.max()) + 1
    totals = np.zeros(k)
    original = [set(np.where(labels == c)[0].tolist()) for c in range(k)]
    for _ in range(n_boot):
        idx = rng.integers(0, n, n)
        km = KMeans(n_clusters=k, random_state=SEED, n_init=10).fit(X[idx])
        recovered = km.predict(X)
        for c in range(k):
            best = 0.0
            for c2 in range(k):
                new = set(np.where(recovered == c2)[0].tolist())
                union = original[c] | new
                if union:
                    best = max(best, len(original[c] & new) / len(union))
```

```
            totals[c] += best
    return totals / n_boot


def cluster_analysis(d: pd.DataFrame, outdir: Path) -> dict:
    X = StandardScaler().fit_transform(d[CLUSTER_VARS].values.astype(float))

    sil = {}
    for k in range(2, 7):
        km = KMeans(n_clusters=k, random_state=SEED, n_init=10).fit(X)
        sil[k] = float(silhouette_score(X, km.labels_))

    km2 = KMeans(n_clusters=2, random_state=SEED, n_init=10).fit(X)
    raw = km2.labels_
    # Label the larger cluster "1" so that the numbering is reproducible and
    # independent of k-means centroid ordering.
    big = int(np.bincount(raw).argmax())
    cluster = np.where(raw == big, 1, 2)
    d = d.assign(cluster=cluster)

    ct = pd.crosstab(d["cluster"], d["sex"])
    chi2 = stats.chi2_contingency(ct, correction=True)[0]
    cramers_v = float(np.sqrt(chi2 / len(d)))
    fisher_p = float(stats.fisher_exact(ct.values)[1])
    jac = jaccard_stability(X, raw)
    jac_ordered = [jac[big], jac[1 - big]]
    ari_published = adjusted_rand_score(d["cluster_published"], d["cluster"])

    # Agreement of the two-cluster solution with a reduced variable set
    # restricted to the six skinfold-corrected girths.
    Xr = StandardScaler().fit_transform(d[CG6].values.astype(float))
    lab_r = KMeans(n_clusters=2, random_state=SEED, n_init=10).fit(Xr).labels_
    ari_reduced = adjusted_rand_score(cluster, lab_r)

    # --- Table A1 ------------------------------------------------------------
    c1, c2 = d[d.cluster == 1], d[d.cluster == 2]
    rows = []
    for var, label, dec in TABLE1_ROWS:
        p = stats.mannwhitneyu(c1[var], c2[var], alternative="two-sided").pvalue
        rows.append({
            "Variable": label,
            f"Cluster 1 (n = {len(c1)})": median_iqr(c1[var], dec),
            f"Cluster 2 (n = {len(c2)})": median_iqr(c2[var], dec),
            "PS": f"{probability_of_superiority(c1[var], c2[var]):.2f}",
            "p": fmt_p(p),
        })
    tA1 = pd.DataFrame(rows)
    tA1.to_csv(outdir / "tableA1_cluster_descriptives.csv", index=False)

    # --- Table A2 ------------------------------------------------------------
    tA2 = pd.DataFrame([
        ("Mean silhouette width (k = 2)", f"{sil[2]:.3f}"),
        ("Mean silhouette width (k = 3)", f"{sil[3]:.3f}"),
        ("Mean silhouette width (k = 4)", f"{sil[4]:.3f}"),
        ("Bootstrap Jaccard stability, cluster 1", f"{jac_ordered[0]:.2f}"),
        ("Bootstrap Jaccard stability, cluster 2", f"{jac_ordered[1]:.2f}"),
        ("Cramer's V with sex", f"{cramers_v:.2f}"),
        ("Fisher's exact test against sex", fmt_p(fisher_p)),
        ("Adjusted Rand index, reduced variable set (six corrected girths)",
         f"{ari_reduced:.2f}"),
    ], columns=["Metric", "Value"])
    tA2.to_csv(outdir / "tableA2_cluster_validity.csv", index=False)

    log("=" * 78)
    log("EXPLORATORY UNSUPERVISED ANALYSIS (Section 3.6; Tables A1-A2, Fig. A1)")
    log("=" * 78)
    log(f"Variables entered ({len(CLUSTER_VARS)}): {', '.join(CLUSTER_VARS)}")
    log(f"Standardisation      : z-scores (mean 0, SD 1)")
    log(f"Algorithm            : k-means, n_init = 10, random_state = {SEED}")
    log("")
    log("Mean silhouette width by number of clusters:")
    for k, s in sil.items():
        log(f"  k = {k}: {s:.3f}")
    log("")
    log(f"Two-cluster solution : n = {len(c1)} and n = {len(c2)}")
    log(f"Agreement with the partition stored in the dataset (ARI): "
        f"{ari_published:.2f}")
    log("Cross-tabulation with sex (rows = cluster, columns = 1 men, 2 women):")
    log(ct.to_string())
    log(f"  Cramer's V         : {cramers_v:.2f}")
    log(f"  Fisher exact p     : {fmt_p(fisher_p)}")
    log(f"  Jaccard stability  : cluster 1 = {jac_ordered[0]:.2f}, "
        f"cluster 2 = {jac_ordered[1]:.2f} ({N_BOOT_STABILITY} resamples)")
    log(f"  ARI, reduced set   : {ari_reduced:.2f}")
    log("")
    log("  Interpretation: the mean silhouette width is well below the value")
    log("  conventionally taken to indicate reasonable separation, and the")
    log("  partition corresponds very largely to sex. The clusters are a")
    log("  sample-derived restatement of the sex difference in body size and")
    log("  are not treated as morphophysiological phenotypes.")
    log("")
    log("TABLE A1 - Descriptive characteristics by cluster (exploratory)")
    log("-" * 78)
    log(tA1.to_string(index=False))
    log("")
    log("TABLE A2 - Internal validity and stability of the cluster solution")
    log("-" * 78)
    log(tA2.to_string(index=False))
    log("")

    # --- Figure A1 -----------------------------------------------------------
    fig, axes = plt.subplots(1, 3, figsize=(10.5, 3.5))
    ks = list(sil)
    axes[0].plot(ks, [sil[k] for k in ks], marker="o", color=OI["blue"], lw=1.6)
    axes[0].axhline(0.50, color=OI["vermil"], ls="--", lw=1.0)
    axes[0].text(ks[-1], 0.51, "Reasonable separation", color=OI["vermil"],
                 fontsize=7, ha="right", va="bottom")
    axes[0].set_xticks(ks)
    axes[0].set_xlabel("Number of clusters (k)")
```

```
    axes[0].set_ylabel("Mean silhouette width")
    axes[0].set_ylim(0, 0.60)
    axes[0].set_title("(A) Internal validity", loc="left", fontweight="bold")

    pcs = PCA(n_components=2, random_state=SEED).fit(X)
    scores = pcs.transform(X)
    for cl, col, mark in [(1, OI["blue"], "o"), (2, OI["green"], "s")]:
        sel = d.cluster.values == cl
        axes[1].scatter(scores[sel, 0], scores[sel, 1], s=26, color=col,
                        marker=mark, label=f"Cluster {cl}", zorder=2)
    fem = d.sex.values == 2
    axes[1].scatter(scores[fem, 0], scores[fem, 1], s=90, facecolors="none",
                    edgecolors=OI["orange"], linewidths=1.1, label="Women",
                    zorder=3)
    axes[1].set_xlabel(f"PC1 ({pcs.explained_variance_ratio_[0] * 100:.1f}% of variance)")
    axes[1].set_ylabel(f"PC2 ({pcs.explained_variance_ratio_[1] * 100:.1f}% of variance)")
    axes[1].set_title("(B) Two-cluster solution", loc="left", fontweight="bold")
    axes[1].legend(loc="best", fontsize=7)

    men_counts = [int(((d.cluster == c) & (d.sex == 1)).sum()) for c in (1, 2)]
    wom_counts = [int(((d.cluster == c) & (d.sex == 2)).sum()) for c in (1, 2)]
    xpos = np.arange(2)
    axes[2].bar(xpos, men_counts, 0.55, color=OI["blue"], label="Men")
    axes[2].bar(xpos, wom_counts, 0.55, bottom=men_counts, color=OI["orange"],
                label="Women")
    for i in range(2):
        if men_counts[i]:
            axes[2].text(i, men_counts[i] / 2, str(men_counts[i]), ha="center",
                         va="center", color="white", fontsize=8)
        if wom_counts[i]:
            axes[2].text(i, men_counts[i] + wom_counts[i] / 2, str(wom_counts[i]),
                         ha="center", va="center", color="black", fontsize=8)
    axes[2].set_xticks(xpos)
    axes[2].set_xticklabels(["Cluster 1", "Cluster 2"])
    axes[2].set_ylabel("Participants (n)")
    axes[2].set_title("(C) Sex composition", loc="left", fontweight="bold")
    axes[2].legend(loc="upper right", fontsize=8)

    fig.tight_layout()
    fig.savefig(outdir / "figureA1_cluster_diagnostics.png", dpi=DPI)
    plt.close(fig)

    return {"sil2": sil[2], "sil3": sil[3], "n1": len(c1), "n2": len(c2),
            "women_c2": int(((d.cluster == 2) & (d.sex == 2)).sum()),
            "cramers_v": cramers_v, "ari_published": float(ari_published),
            "jaccard1": float(jac_ordered[0]), "jaccard2": float(jac_ordered[1])}


# ============================================================================
# 12. REPRODUCTION CHECK
# ============================================================================

def reproduction_check(computed: dict, outdir: Path) -> pd.DataFrame:
    rows = []
    for label, published, tol in PUBLISHED:
        got = computed.get(label)
        if got is None:
            rows.append({"Quantity": label, "Published": published,
                         "Computed": "not computed", "Tolerance": tol,
                         "Kind": "stochastic" if label in STOCHASTIC else "deterministic",
                         "Status": "SKIPPED"})
            continue
        ok = abs(float(got) - float(published)) <= tol + 1e-12
        rows.append({"Quantity": label, "Published": published,
                     "Computed": round(float(got), 4),
                     "Tolerance": tol,
                     "Kind": "stochastic" if label in STOCHASTIC else "deterministic",
                     "Status": "MATCH" if ok else "DIFFERS"})
    chk = pd.DataFrame(rows)
    chk.to_csv(outdir / "reproduction_check.csv", index=False)

    n_ok = int((chk.Status == "MATCH").sum())
    log("=" * 78)
    log("REPRODUCTION CHECK - computed values against the published article")
    log("=" * 78)
    log(chk.to_string(index=False))
    log("")
    log(f"{n_ok} of {len(chk)} checked quantities reproduce within tolerance.")
    log("")
    return chk



# ============================================================================
# 13. SESSION INFORMATION
# ============================================================================

def session_info(outdir: Path) -> str:
    lines = [
        "SESSION INFORMATION",
        "=" * 78,
        f"Python             : {sys.version.split()[0]}",
        f"Platform           : {platform.platform()}",
        f"numpy              : {np.__version__}",
        f"pandas             : {pd.__version__}",
        f"scipy              : {scipy.__version__}",
        f"statsmodels        : {statsmodels.__version__}",
        f"scikit-learn       : {sklearn.__version__}",
        f"matplotlib         : {matplotlib.__version__}",
        f"Random seed        : {SEED}",
        f"Bootstrap resamples : {N_BOOT} (correlations, coefficients); "
        f"{N_BOOT_STABILITY} (cluster stability)",
        "=" * 78,
    ]
    text = "\n".join(lines)
    (outdir / "session_info.txt").write_text(text + "\n")
    log(text)
    log("")
    return text
```

```
# ==============================================================================
# 14. MAIN
# ==============================================================================

def main() -> None:
    ap = argparse.ArgumentParser(
        description="Reproduce the analyses of the MFO manuscript.")
    ap.add_argument("--data", default="Dataset_MFO.xlsx",
                    help="Path to the analytic dataset (.xlsx)")
    ap.add_argument("--outdir", default="outputs",
                    help="Directory for tables, figures and logs")
    args = ap.parse_args()

    outdir = Path(args.outdir)
    outdir.mkdir(parents=True, exist_ok=True)

    np.random.seed(SEED)  # legacy global seed, for completeness

    d = load_data(Path(args.data))

    data_integrity_checks(d, outdir)
    table1(d, outdir)
    table2_figure2(d, outdir)
    prim = primary_model(d, outdir)
    table3_figure4(d, prim["aic"], outdir)
    table4(d, float(prim["res"].rsquared), outdir)
    norm = normalised_specification(d, outdir)
    table5(d, outdir)
    table6_figureA2(outdir)
    clus = cluster_analysis(d, outdir)

    # ---- assemble the quantities that are checked against the article -------
    res = prim["res"]
    men, women = d[d.sex == 1], d[d.sex == 2]
    r_men, _, y_men = fit_ols(men, ["vo2_abs"])
    r_wom, _, y_wom = fit_ols(women, ["vo2_abs"])
    s1, _, y = fit_ols(d, ["vo2_abs", "calf_cg"])
    s7, _, _ = fit_ols(d, ["vo2_abs", "ffm_dxa"])
    s5, _, _ = fit_ols(d, ["vo2_abs", "calf_cg", "ffm_dxa"])
    s11, _, y11 = fit_ols(d, ["vo2_abs", "calf_cg"], outcome="mfo_per_ffm")
    norm2, _, _ = fit_ols(d, ["vo2_rel", "calf_cg"])

    computed = {
        "n (analytic sample)": len(d),
        "n men": len(men),
        "n women": len(women),
        "Median MFO (g/min)": float(d.mfo.median()),
        "Median absolute VO2peak (L/min)": float(d.vo2_abs.median()),
        "Median normalised VO2peak (mL/kg/min)": float(d.vo2_rel.median()),
        "Spearman rho, MFO ~ absolute VO2peak":
            float(stats.spearmanr(d.mfo, d.vo2_abs).statistic),
        "Spearman rho, MFO ~ normalised VO2peak":
            float(stats.spearmanr(d.mfo, d.vo2_rel).statistic),
        "Spearman rho, MFO ~ corrected calf girth":
            float(stats.spearmanr(d.mfo, d.calf_cg).statistic),
        "M1 R2": float(res.rsquared),
        "M1 adjusted R2": float(res.rsquared_adj),
        "M1 Q2 (LOOCV)": prim["q2"],
        "M1 F statistic": float(res.fvalue),
        "M1 beta, absolute VO2peak": float(res.params[1]),
        "M1 AIC": float(res.aic),
        "M1 Breusch-Pagan p": prim["bp_p"],
        "M1 RESET p": prim["reset_p"],
        "M1 Durbin-Watson": prim["dw"],
        "M1 Shapiro-Wilk p (residuals)": prim["sw_p"],
        "M1 n Cook's D above 4/n": prim["n_infl"],
        "Allometric scaling exponent": prim["allo_exp"],
        "S1 delta R2 (corrected calf girth)":
            float(s1.rsquared) - float(res.rsquared),
        "S1 beta (corrected calf girth)": float(s1.params[2]),
        "S1 p (corrected calf girth)": float(s1.pvalues[2]),
        "S1 Q2": loocv_q2(s1, y),
        "S1 delta AIC": float(s1.aic) - float(res.aic),
        "S7 beta (fat-free mass, DXA)": float(s7.params[2]),
        "S7 p (fat-free mass, DXA)": float(s7.pvalues[2]),
        "S5 p (corrected calf girth)": float(s5.pvalues[2]),
        "S5 p (fat-free mass)": float(s5.pvalues[3]),
        "S11 R2 (outcome scaled to FFM)": float(s11.rsquared),
        "S11 Q2 (outcome scaled to FFM)": loocv_q2(s11, y11),
        "Normalised specification R2": norm["r2_norm"],
        "Normalised specification Q2": norm["q2_norm"],
        "Normalised specification AIC": norm["aic_norm"],
        "Normalised + calf girth, beta": float(norm2.params[2]),
        "Normalised + calf girth, R2": float(norm2.rsquared),
        "Men: R2": float(r_men.rsquared),
        "Men: Q2": loocv_q2(r_men, y_men),
        "Men: beta": float(r_men.params[1]),
        "Women: R2": float(r_wom.rsquared),
        "Women: Q2": loocv_q2(r_wom, y_wom),
        "Women: beta": float(r_wom.params[1]),
        "Women: p": float(r_wom.pvalues[1]),
        "Mean silhouette width (k = 2)": clus["sil2"],
        "Mean silhouette width (k = 3)": clus["sil3"],
        "Cluster 1 size": clus["n1"],
        "Cluster 2 size": clus["n2"],
        "Women in cluster 2": clus["women_c2"],
        "Cramer's V, cluster vs sex": clus["cramers_v"],
        "Bootstrap Jaccard stability, cluster 1": clus["jaccard1"],
        "Bootstrap Jaccard stability, cluster 2": clus["jaccard2"],
    }
    reproduction_check(computed, outdir)
    session_info(outdir)

    (outdir / "analysis_log.txt").write_text("\n".join(LOG_LINES) + "\n")
    print(f"\nAll outputs written to: {outdir.resolve()}")
```

```
if __name__ == "__main__":
    main()
```
